# Supplementary material for: Cancer-wide in silico analyses using differentially expressed genes demonstrate the functions and clinical relevance of JAG, DLL, and NOTCH
Source: PLoS One. 2024 Jul 29;19(7):e0307943. doi: 10.1371/journal.pone.0307943 (PMC11285958; doi:10.1371/journal.pone.0307943)
Supplement: S1 File — (PDF) [file pone.0307943.s001.pdf]

Cancer-wide *In Silico* Analyses Using the Differentially Expressed Genes Demonstrate the Functions and Clinical Relevance of *JAG*, *DLL*, and *NOTCH*

Jung Yun Kim[1,2], Nayoung Hong[1,2], Seok Won Ham[3], Sehyeon Park[1,2], Sunyoung Seo[1,2] and Hyunggee Kim[*,1,2]

[1]Department of Biotechnology, College of Life Sciences and Biotechnology, Korea University, Seoul, Republic of Korea

[2]Institute of Animal Molecular Biotechnology, Korea University, Seoul, Republic of Korea

[3]MEDIFIC Inc., Hwaseong-si, Gyeonggi-do, Republic of Korea

[*]Corresponding author

E-mail: hg-kim@korea.ac.kr

A

Cancer types	Sources	Gene counts	Patient No.
Bladder urothelial carcinoma (BUC)	TCGA, Provisional (cBioPortal)	20,437	407
Breast invasive carcinoma (BIC)	TCGA, Provisional (cBioPortal)	20,437	1,099
Colorectal adenocarcinoma (CRC)	TCGA, Provisional (cBioPortal)	20,437	377
Glioblastoma multiforme (GBM)	TCGA, Provisional (GlioVis)	12,701	538
Head and neck squamous cell carcinoma (HNSC)	TCGA, Provisional (cBioPortal)	20,437	519
Kidney renal clear cell carcinoma (KRCC)	TCGA, Provisional (cBioPortal)	20,437	532
Low grade glioma (LGG)	TCGA, Provisional (cBioPortal)	20,437	528
Liver hepatocellular carcinoma (LHC)	TCGA, Provisional (cBioPortal)	20,437	370
Lung adenocarcinoma (LUAD)	TCGA, Provisional (cBioPortal)	20,466	223
Ovarian serous cystadenocarcinoma (OV)	TCGA, Provisional (cBioPortal)	20,437	307
Prostate adenocarcinoma (PRAD)	TCGA, Provisional (cBioPortal)	20,437	497
Skin cutaneous melanoma (SCC)	TCGA, Provisional (cBioPortal)	20,437	471
Stomach adenocarcinoma (STAD)	TCGA, Provisional (cBioPortal)	20,437	414
Thyroid carcinoma (TC)	TCGA, Provisional (cBioPortal)	20,437	500
Uterine corpus endometrial carcinoma (UCEC)	TCGA, Provisional (cBioPortal)	20,437	176

B

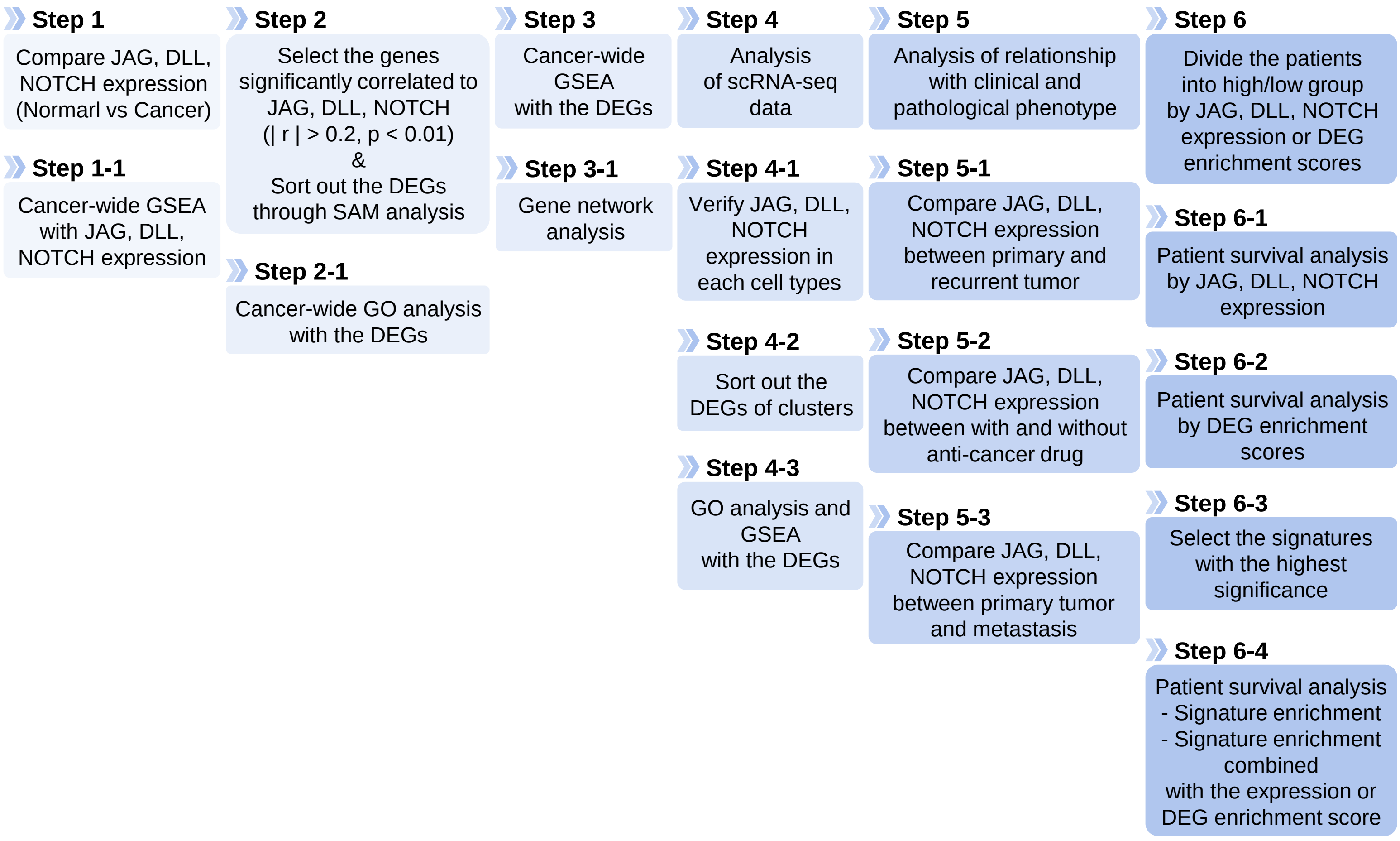

S1 Fig. Information of the clinical datasets and overall scheme of our study.

(A) A table showing the information of the datasets, such as sources, gene counts, and the number of patients included in the dataset.

(B) Workflow of the overall bioinformatic analysis.

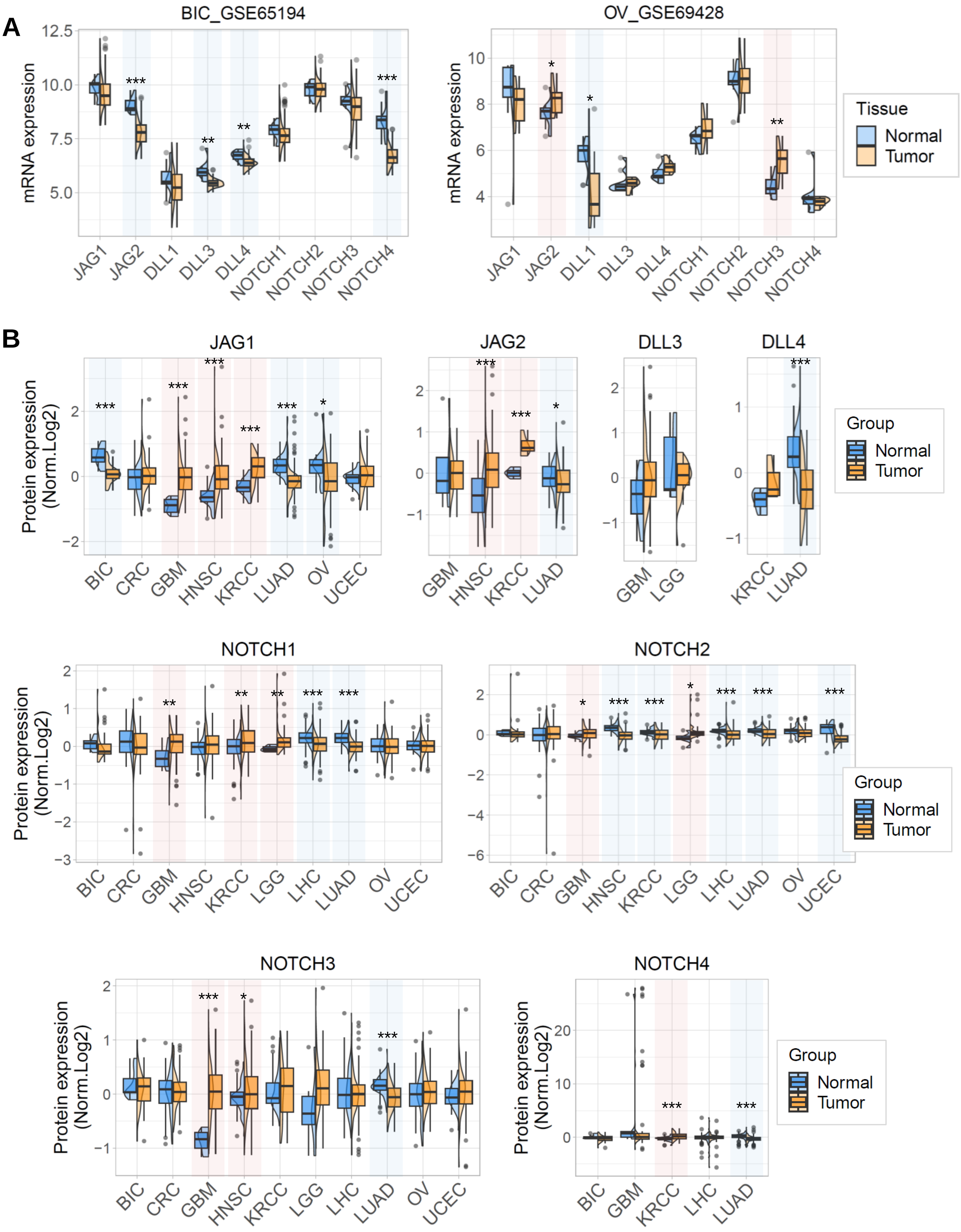
A
B
BIC_GSE65194
OV_GSE69428
mRNA expression
Tissue
Normal
Tumor
JAG1
JAG2
DLL1
DLL3
DLL4
NOTCH1
NOTCH2
NOTCH3
NOTCH4
Protein expression (Norm.Log2)
Group
BIC
CRC
GBM
HNSC
KRCC
LGG
LHC
LUAD
OV
UCEC

S2 Fig. JAG, DLL, and NOTCH families' mRNA and protein expression.

(A) Box plots comparing the gene expressions of the JAG, DLL, and NOTCH families in BIC (GSE65194) and OV (GSE69428) and their corresponding normal tissues. Data were analyzed using a two-tailed Student's t-test (*$p < 0.05$, **$p < 0.01$, ***$p < 0.001$).

(B) Box plots comparing the protein expressions of the JAG, DLL, and NOTCH families in BIC, CRC, GBM, HNSC, KRCC, LHC, LGG, LUAD, OV, and UCEC and their corresponding normal tissues. Data were analyzed using a two-tailed Student's t-test (*$p < 0.05$; **$p < 0.01$; ***$p < 0.001$).

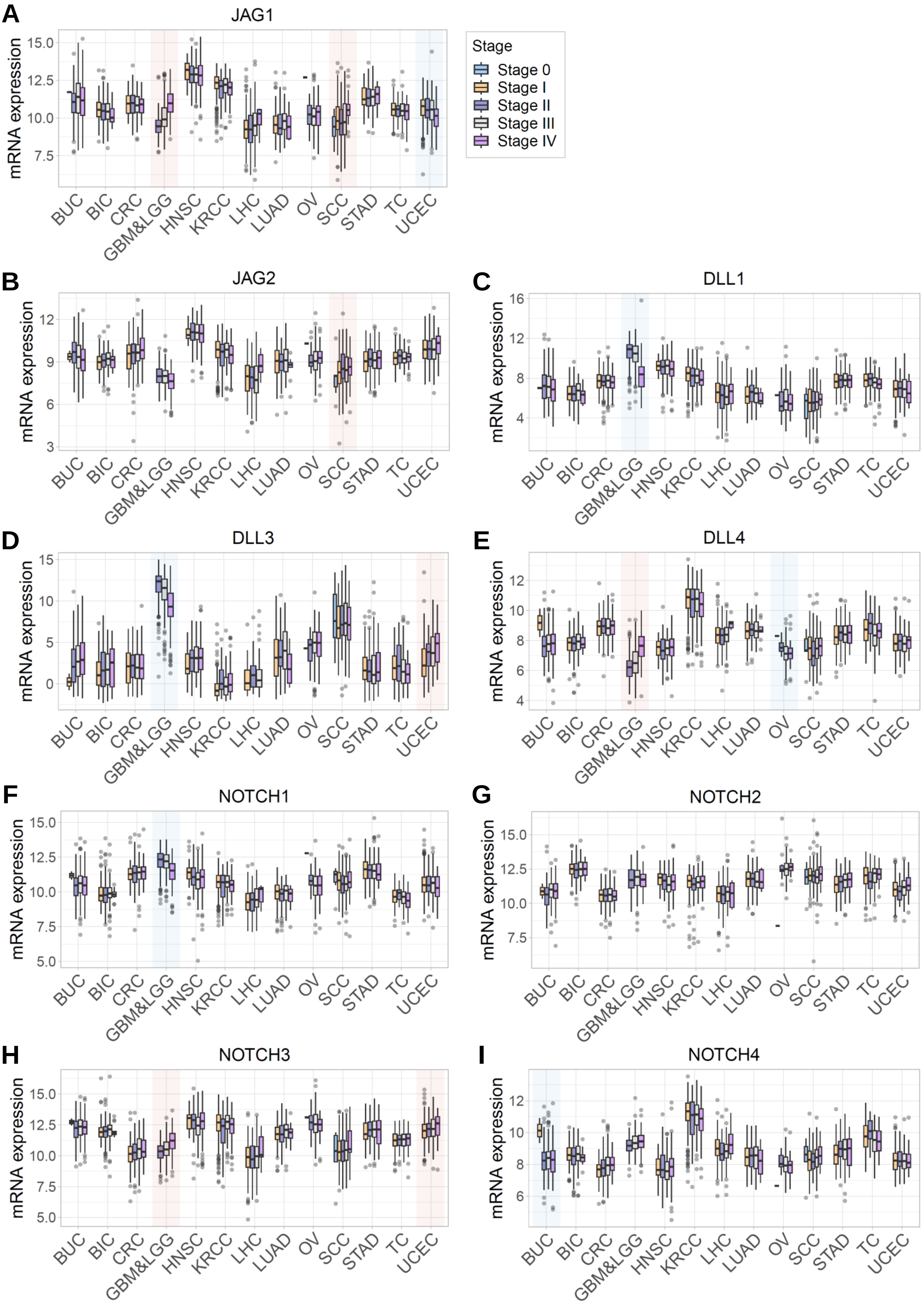
A
JAG1
B
JAG2
C
DLL1
D
DLL3
E
DLL4
F
NOTCH1
G
NOTCH2
H
NOTCH3
I
NOTCH4
mRNA expression
Stage
Stage 0
Stage I
Stage II
Stage III
Stage IV
BUC
BIC
CRC
GBM&LGG
HNSC
KRCC
LHC
LUAD
OV
SCC
STAD
TC
UCEC

S3 Fig. Comparison of mRNA expression of JAG, DLL, and NOTCH families according to tumor stage. Box plots showing the gene expression of (A) *JAG1*, (B) *JAG2*, (C) *DLL1*, (D) *DLL3*, (E) *DLL4*, (F) *NOTCH1*, (G) *NOTCH2*, (H) *NOTCH3*, and (I) *NOTCH4* according to tumor stage.

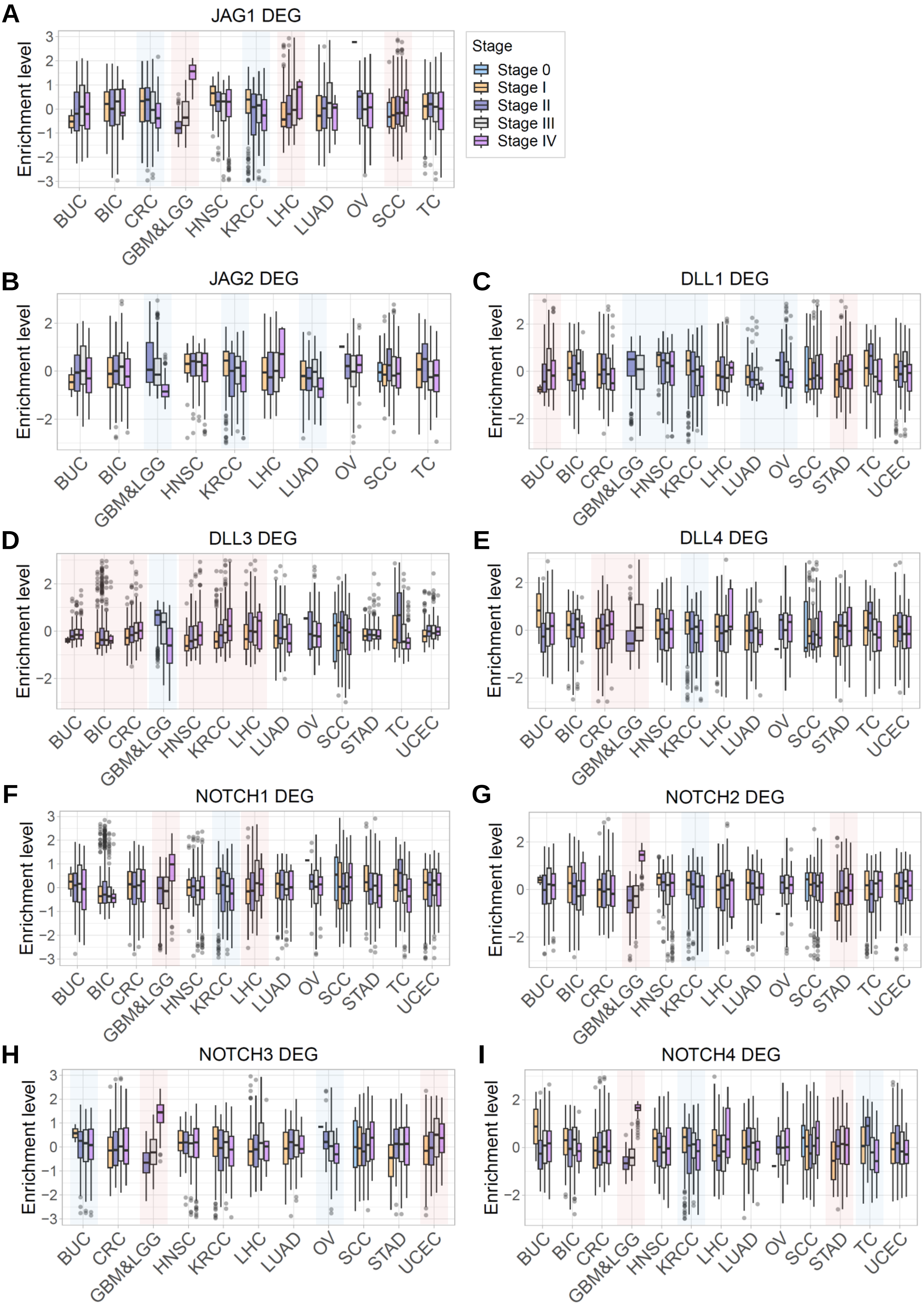
A
JAG1 DEG
Enrichment level
BUC
BIC
CRC
GBM&LGG
HNSC
KRCC
LHC
LUAD
OV
SCC
TC
Stage
Stage 0
Stage I
Stage II
Stage III
Stage IV
B
JAG2 DEG
C
DLL1 DEG
D
DLL3 DEG
E
DLL4 DEG
F
NOTCH1 DEG
G
NOTCH2 DEG
H
NOTCH3 DEG
I
NOTCH4 DEG
STAD
UCEC

S4 Fig. Comparison of enrichment score of DEG for each JAG, DLL, and NOTCH families according to tumor stage. Box plots showing the enrichment score of (A) JAG1 DEG, (B) JAG2 DEG, (C) DLL1 DEG, (D) DLL3 DEG, (E) DLL4 DEG, (F) NOTCH1 DEG, (G) NOTCH2 DEG, (H) NOTCH3 DEG, and (I) NOTCH4 DEG according to tumor stage.

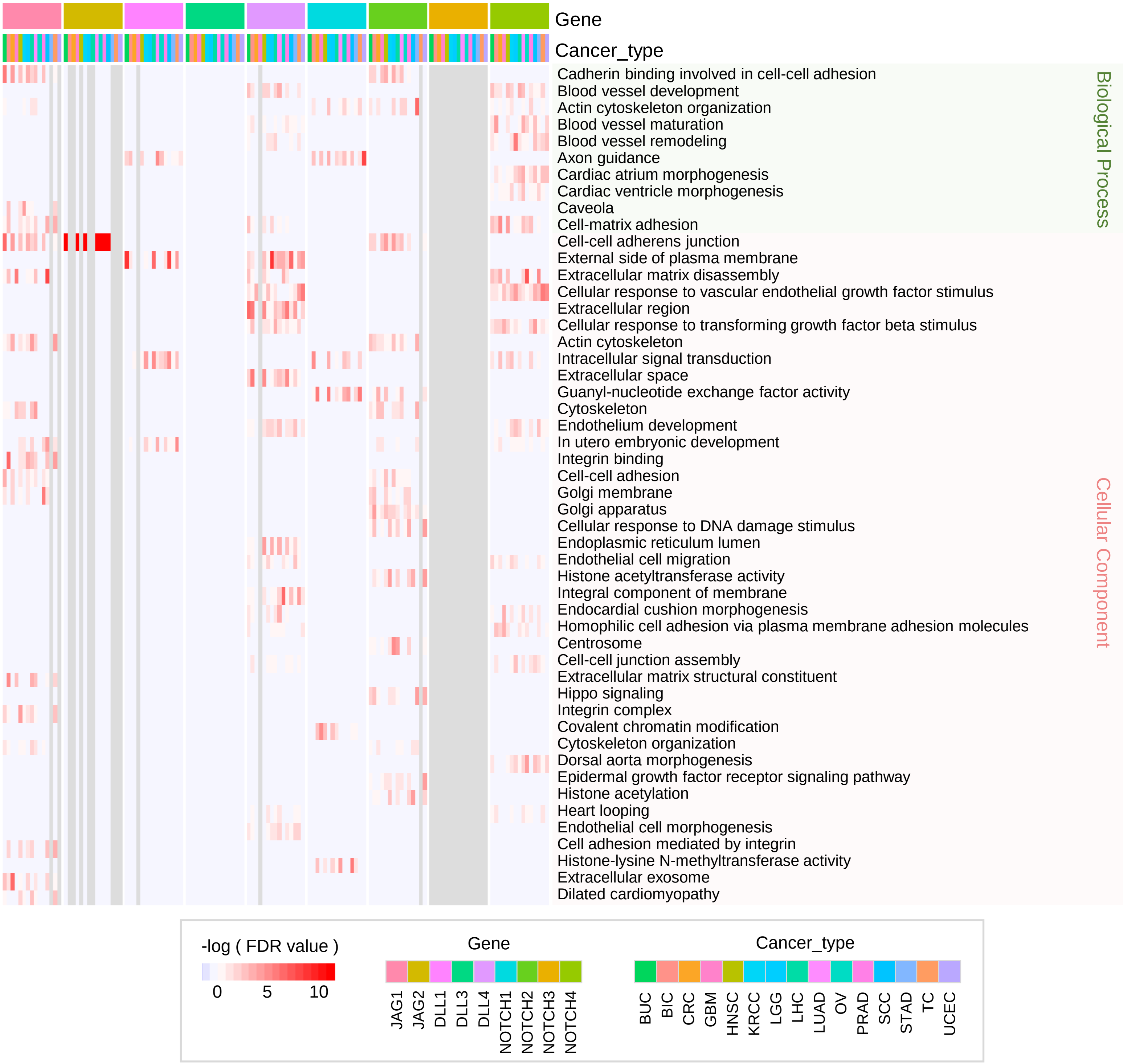

S5 Fig. Heatmap showing the FDR *q*-value of GO analysis results. Each GO term category is included in biological processes and cellular components.

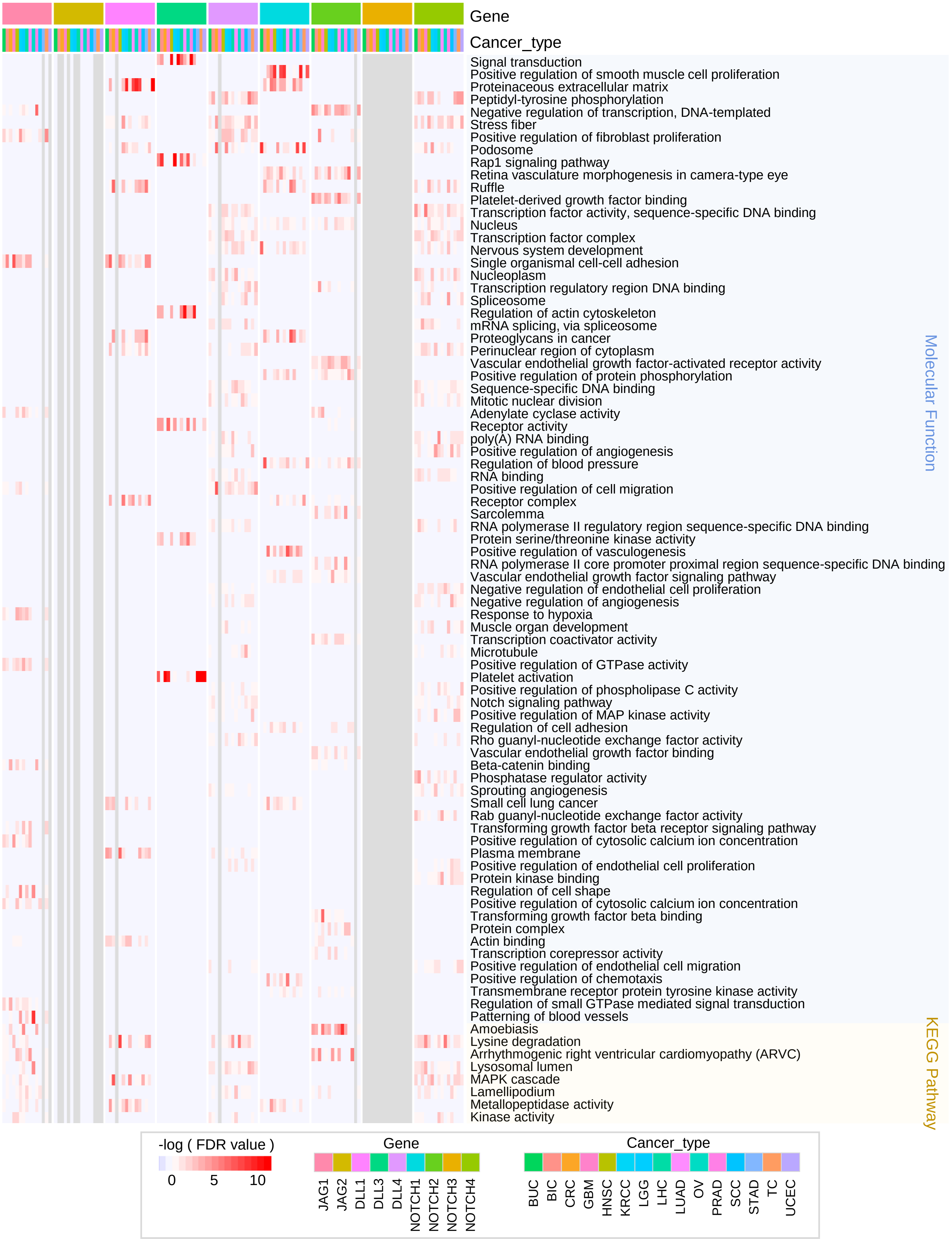

S6 Fig. Heatmap showing the FDR *q*-value of GO analysis results. Each GO term category is included in molecular functions and KEGG pathway.

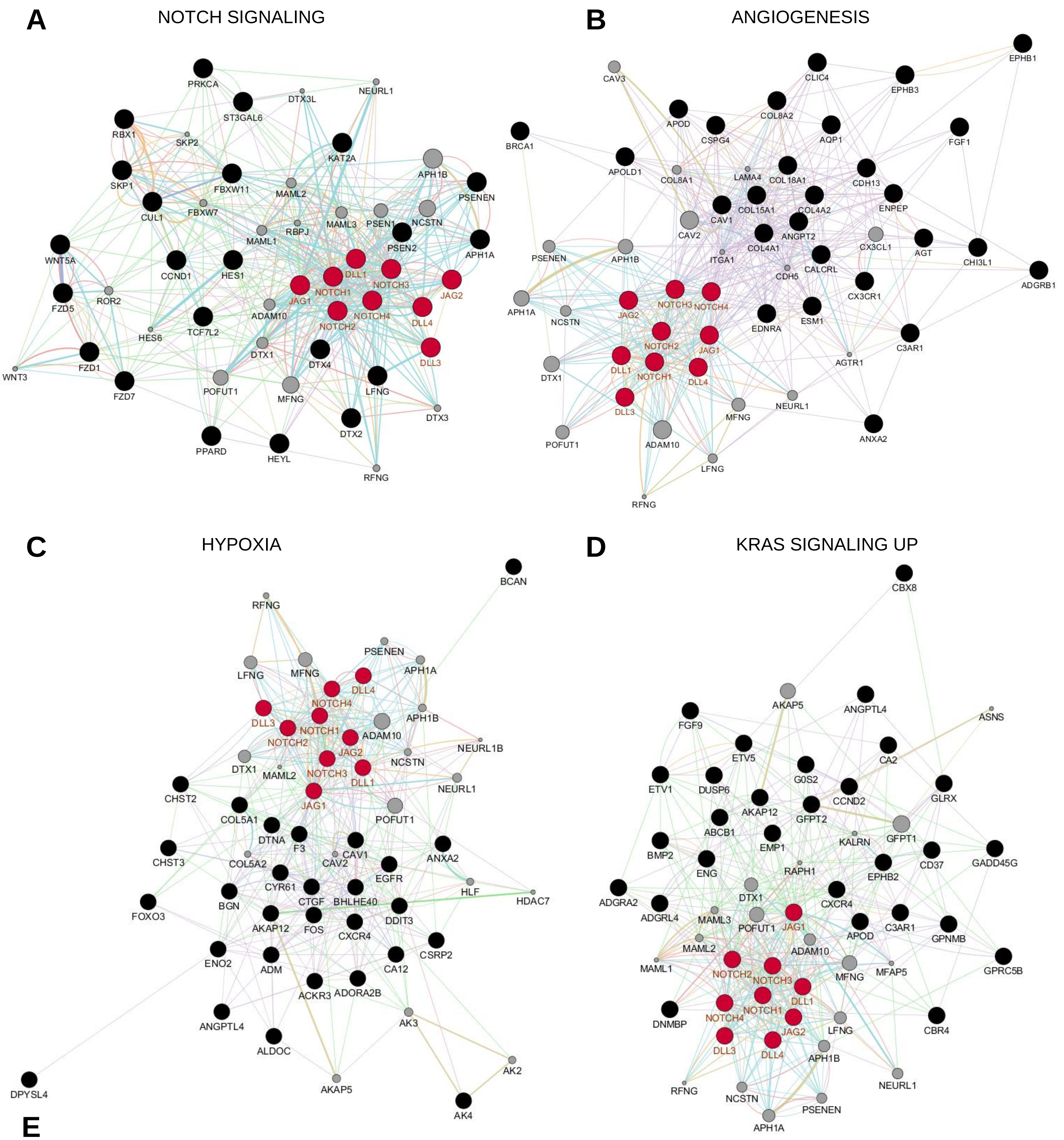

E

Interactions (%)	NOTCH SIGNALING	ANGIOGENESIS	HYPOXIA	KRAS SIGNALING UP
Co-expression	8.78	57.16	41.51	36.33
Physical interactions	23.51	5.79	12.20	14.93
Pathway	37.93	12.05	14.29	20.41
Shared protein domains	4.73	11.84	7.45	13.71
Genetic interactions	3.25	4.10	4.68	4.86
Predicted	14.68	6.40	6.60	6.76
Co-localization	7.12	2.66	13.27	3.00

S7 Fig. Gene network analysis depicts the interactions between JAG, DLL, NOTCH genes and the DEGs, in the regulatory network of the associated signatures. Gene network plots for each signature including (A) Notch signaling, (B) Angiogenesis, (C) Hypoxia, and (D) KRAS signaling UP. Black dots indicate the genes belonging to the DEGs, which are involved in the regulatory network of the signature. Grey dots denote the mediator which are not included in the DEGs but essential for the regulation of the signature. (E) A table showing the compositions of each interaction category described in the gene network analysis.

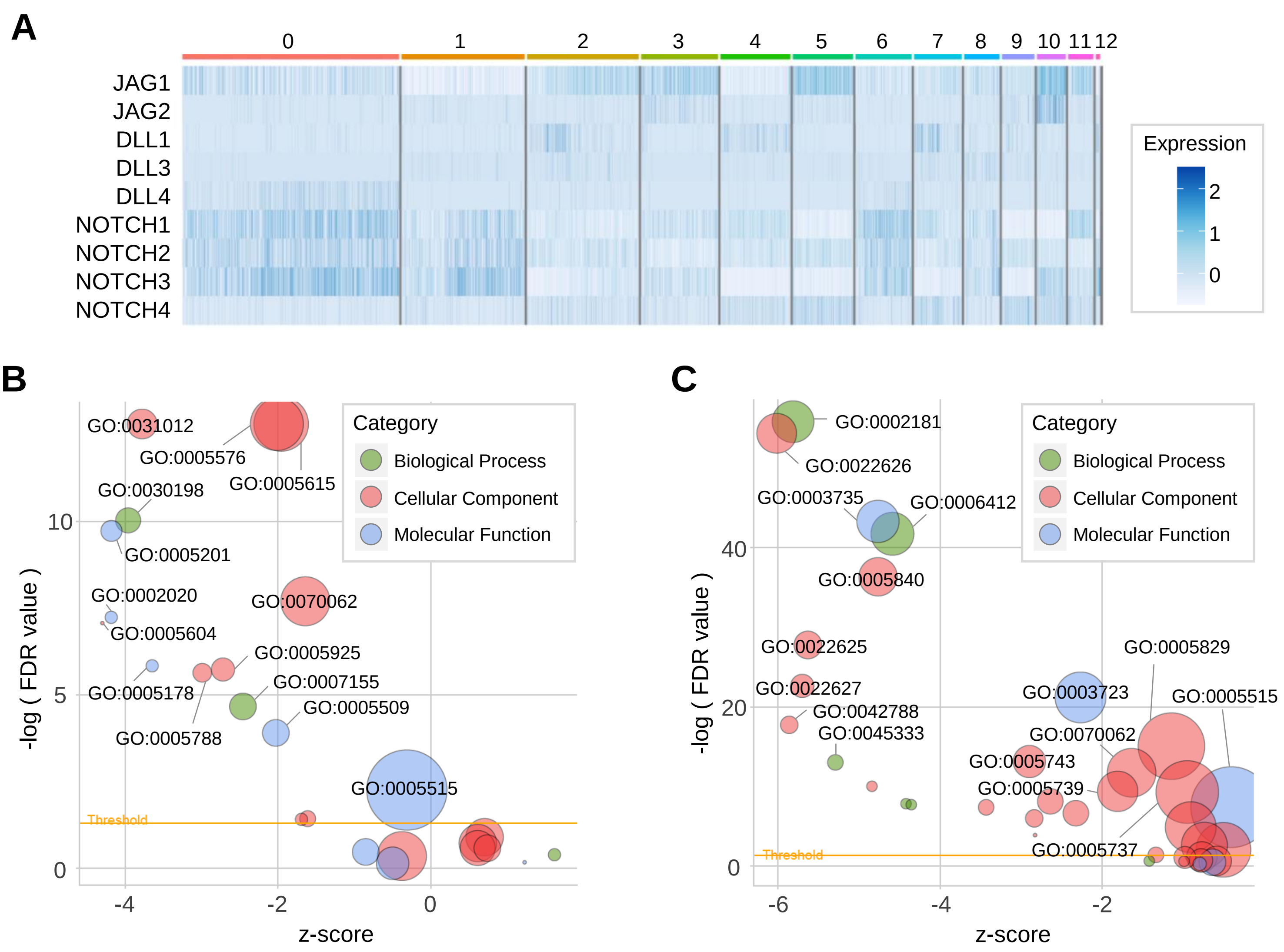

ID	Description
GO:0030198	Extracellular matrix organization
GO:0007155	Cell adhesion
GO:0005576	Extracellular region
GO:0005615	Extracellular space
GO:0031012	Extracellular matrix
GO:0070062	Extracellular exosome
GO:0005604	Basement membrane
GO:0005925	Focal adhesion
GO:0005788	Endoplasmic reticulum lumen
GO:0005201	Extracellular matrix structural constituent
GO:0002020	Protease binding
GO:0005178	Integrin binding
GO:0005509	Calcium ion binding
GO:0005515	Protein binding

ID	Description
GO:0002181	Cytoplasmic translation
GO:0006412	Translation
GO:0045333	Cellular respiration
GO:0022626	Cytosolic ribosome
GO:0005840	Ribosome
GO:0022625	Cytosolic large ribosomal subunit
GO:0022627	Cytosolic small ribosomal subunit
GO:0042788	Polysomal ribosome
GO:0005829	Cytosol
GO:0005743	Mitochondrial inner membrane
GO:0070062	Extracellular exosome
GO:0005739	Mitochondrion
GO:0005737	Cytoplasm
GO:0003735	Structural constituent of ribosome
GO:0003723	RNA binding
GO:0005515	Protein binding

S8 Fig. DEG-based GO analysis using scRNA-seq in GBM.

(A) Heatmap showing the expression of JAG, DLL, and NOTCH families in each cluster.

(B and C) Bubble plot demonstrating GO term enrichment in (B) Cluster 10 and (C) Cluster 0. The bubble size represents the number of DEGs in each GO term. GO terms [-log (FDR value) < 2.5] are listed in the table below.

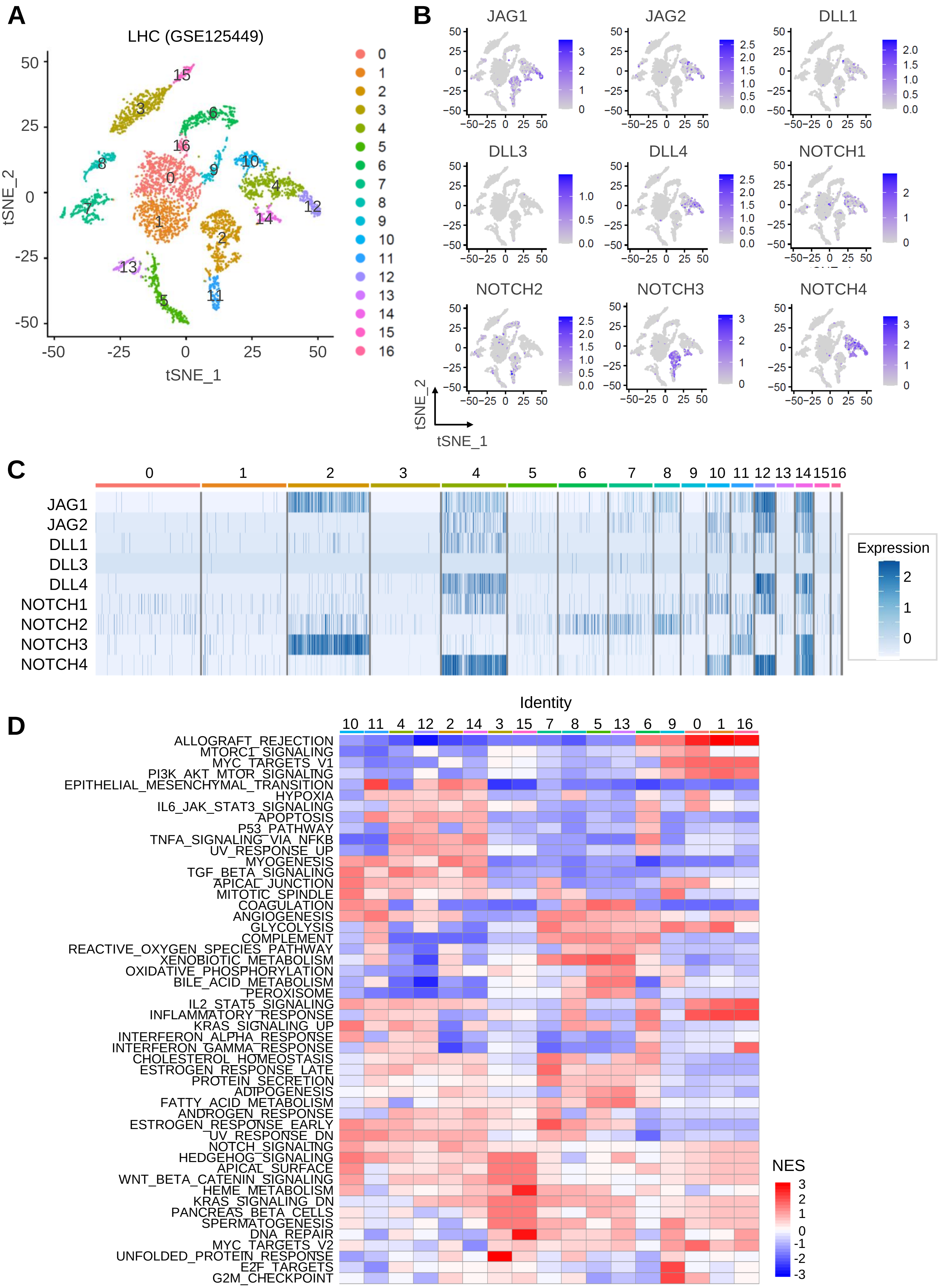
A
LHC (GSE125449)
tSNE_1
tSNE_2
0
1
2
3
4
5
6
7
8
9
10
11
12
13
14
15
16
B
JAG1
JAG2
DLL1
DLL3
DLL4
NOTCH1
NOTCH2
NOTCH3
NOTCH4
tSNE_2
tSNE_1
C
JAG1
JAG2
DLL1
DLL3
DLL4
NOTCH1
NOTCH2
NOTCH3
NOTCH4
Expression
D
Identity
10 11 4 12 2 14 3 15 7 8 5 13 6 9 0 1 16
ALLOGRAFT_REJECTION
MTORC1_SIGNALING
MYC_TARGETS_V1
PI3K_AKT_MTOR_SIGNALING
EPITHELIAL_MESENCHYMAL_TRANSITION
HYPOXIA
IL6_JAK_STAT3_SIGNALING
APOPTOSIS
P53_PATHWAY
TNFA_SIGNALING_VIA_NFKB
UV_RESPONSE_UP
MYOGENESIS
TGF_BETA_SIGNALING
APICAL_JUNCTION
MITOTIC_SPINDLE
COAGULATION
ANGIOGENESIS
GLYCOLYSIS
COMPLEMENT
REACTIVE_OXYGEN_SPECIES_PATHWAY
XENOBIOTIC_METABOLISM
OXIDATIVE_PHOSPHORYLATION
BILE_ACID_METABOLISM
PEROXISOME
IL2_STAT5_SIGNALING
INFLAMMATORY_RESPONSE
KRAS_SIGNALING_UP
INTERFERON_ALPHA_RESPONSE
INTERFERON_GAMMA_RESPONSE
CHOLESTEROL_HOMEOSTASIS
ESTROGEN_RESPONSE_LATE
PROTEIN_SECRETION
ADIPOGENESIS
FATTY_ACID_METABOLISM
ANDROGEN_RESPONSE
ESTROGEN_RESPONSE_EARLY
UV_RESPONSE_DN
NOTCH_SIGNALING
HEDGEHOG_SIGNALING
APICAL_SURFACE
WNT_BETA_CATENIN_SIGNALING
HEME_METABOLISM
KRAS_SIGNALING_DN
PANCREAS_BETA_CELLS
SPERMATOGENESIS
DNA_REPAIR
MYC_TARGETS_V2
UNFOLDED_PROTEIN_RESPONSE
E2F_TARGETS
G2M_CHECKPOINT
NES

S9 Fig. DEG-based GSEA of hallmark signatures using scRNA-seq in LHC (GSE125449).

(A) tSNE plot of LHC in clusters.

(B) Log-normalized expression of JAG, DLL, and NOTCH families.

(C) Heatmap showing the expression of JAG, DLL, and NOTCH families in each cluster.

(D) Heatmap showing the enrichment of hallmark signatures in each cluster.

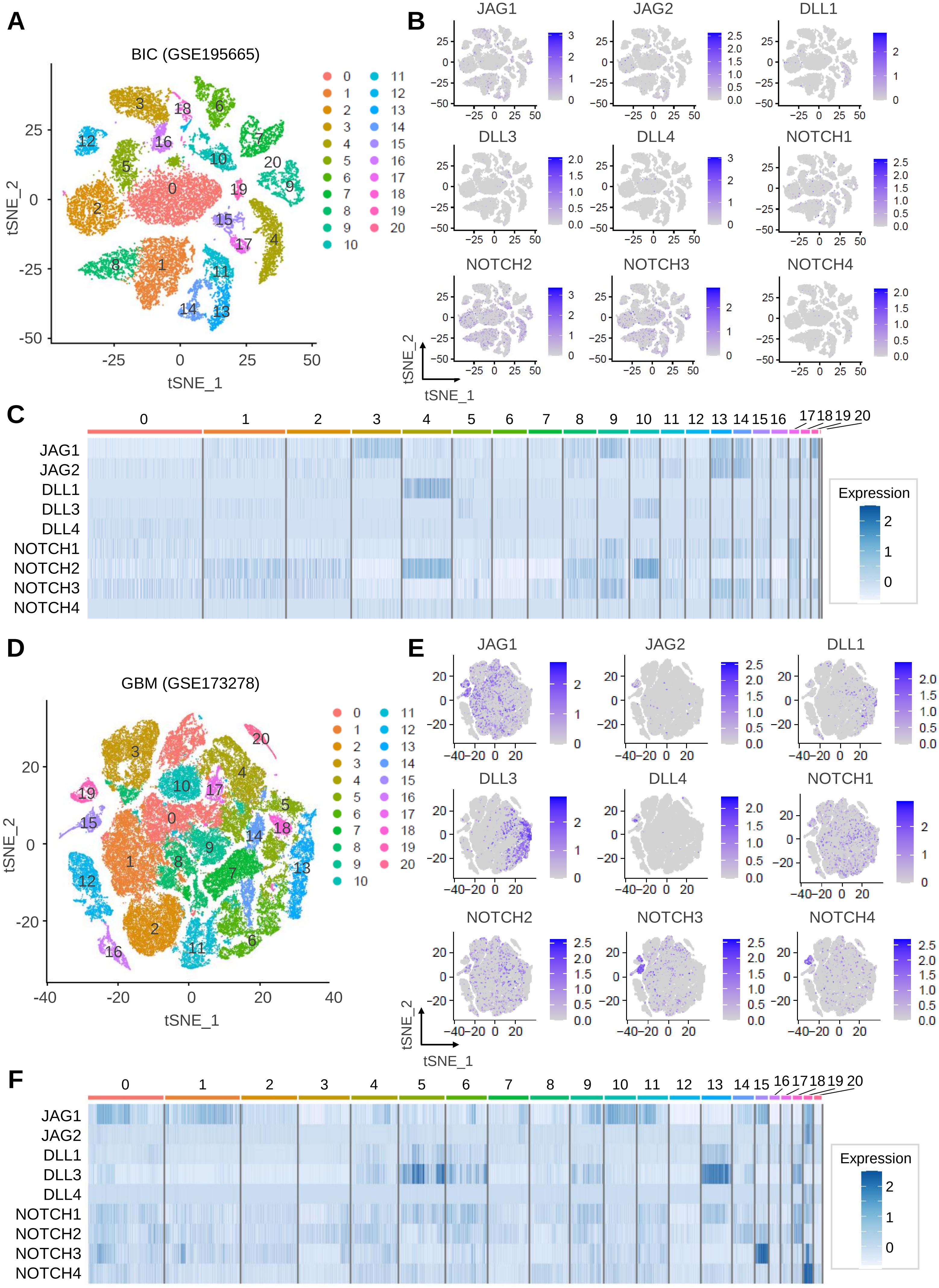
A
BIC (GSE195665)
tSNE_1
tSNE_2
B
JAG1
JAG2
DLL1
DLL3
DLL4
NOTCH1
NOTCH2
NOTCH3
NOTCH4
C
Expression
D
GBM (GSE173278)
E
F

S10 Fig. DEG-based GSEA of hallmark signatures using scRNA-seq in BIC (GSE195665) and GBM (GSE176278).

(A, D) tSNE plot of (A) BIC and (D) GBM in clusters.

(B, E) Log-normalized expression of JAG, DLL, and NOTCH families in (B) BIC and (E) GBM cells.

(C, F) Heatmap showing the expression of JAG, DLL, and NOTCH families in each cluster of (C) BIC and (F) GBM.

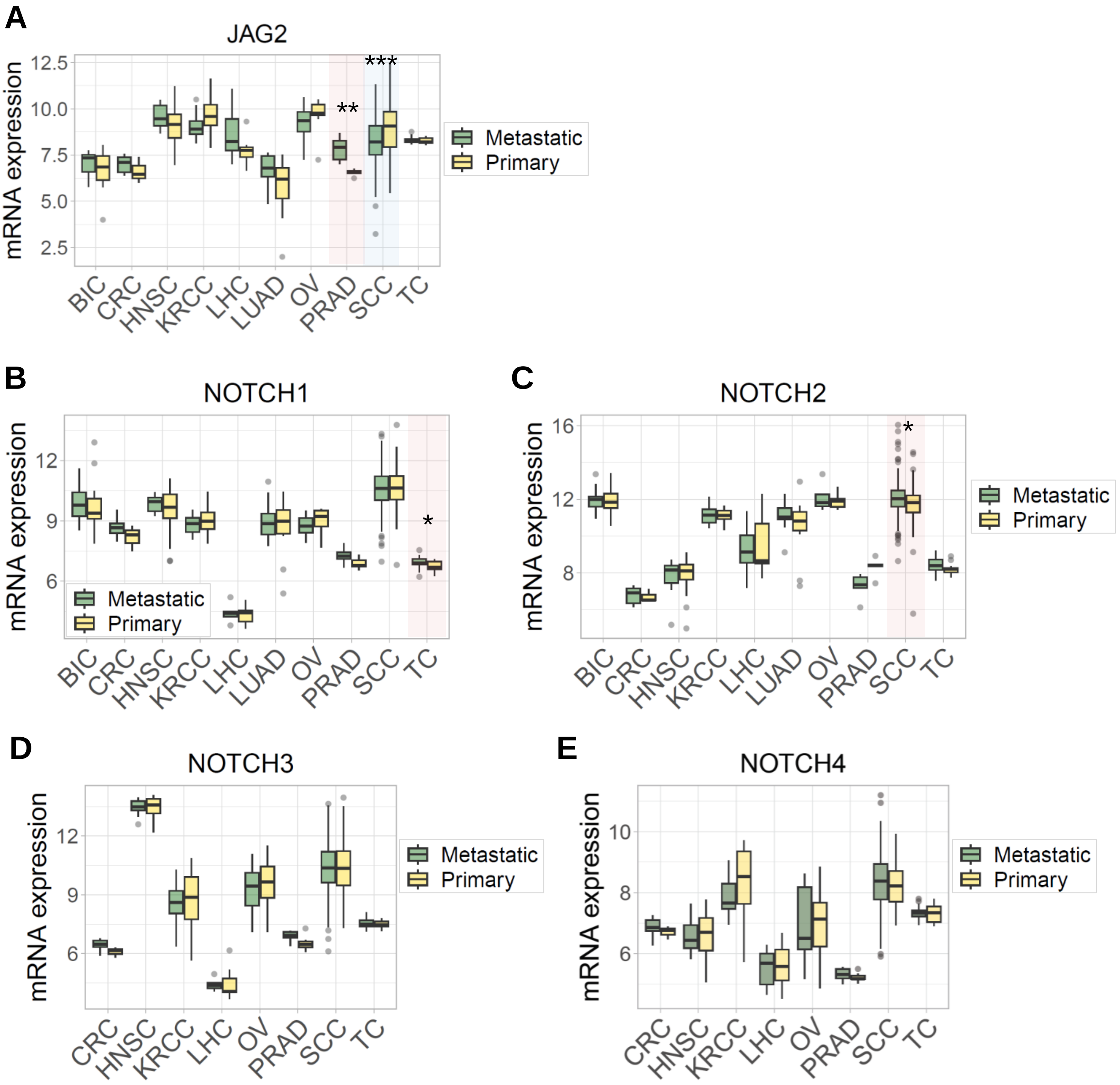

S11 Fig. Box plots showing the expression of (A) *JAG2*, (B) *NOTCH1*, (C) *NOTCH2*, (D) *NOTCH3*, and (E) *NOTCH4* in primary and metastatic tumors. Data were analyzed using a two-tailed Student's t-test (*$p < 0.05$, **$p < 0.01$, ***$p < 0.001$).

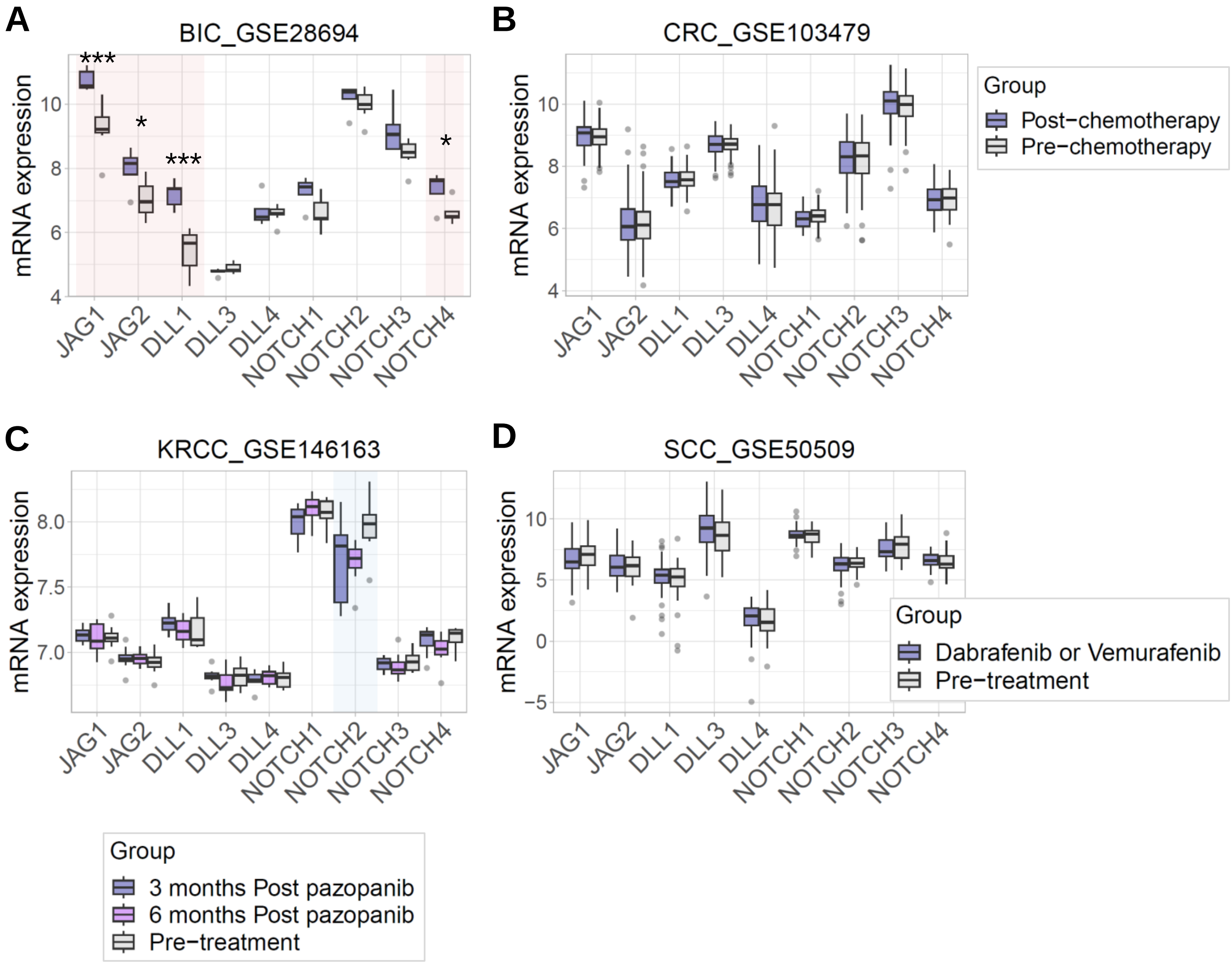

S12 Fig. Box plots comparing the expression of JAG, DLL, and NOTCH families in patients with anti-cancer drugs.

(A) Expression of JAG, DLL, and NOTCH families before and after chemotherapy in BIC. Data were analyzed using a two-tailed Student's t-test (*$p < 0.05$, ***$p < 0.001$).

(B) Expression of JAG, DLL, and NOTCH families before and after chemotherapy in CRC.

(C) Expression of JAG, DLL, and NOTCH families before, 3 months and 6 months after chemotherapy with pazopanib in KRCC. Data were analyzed using a two-tailed Student's t-test.

(D) Expression of JAG, DLL, and NOTCH families before and after chemotherapy with dabrafenib or vemurafenib in SCC.

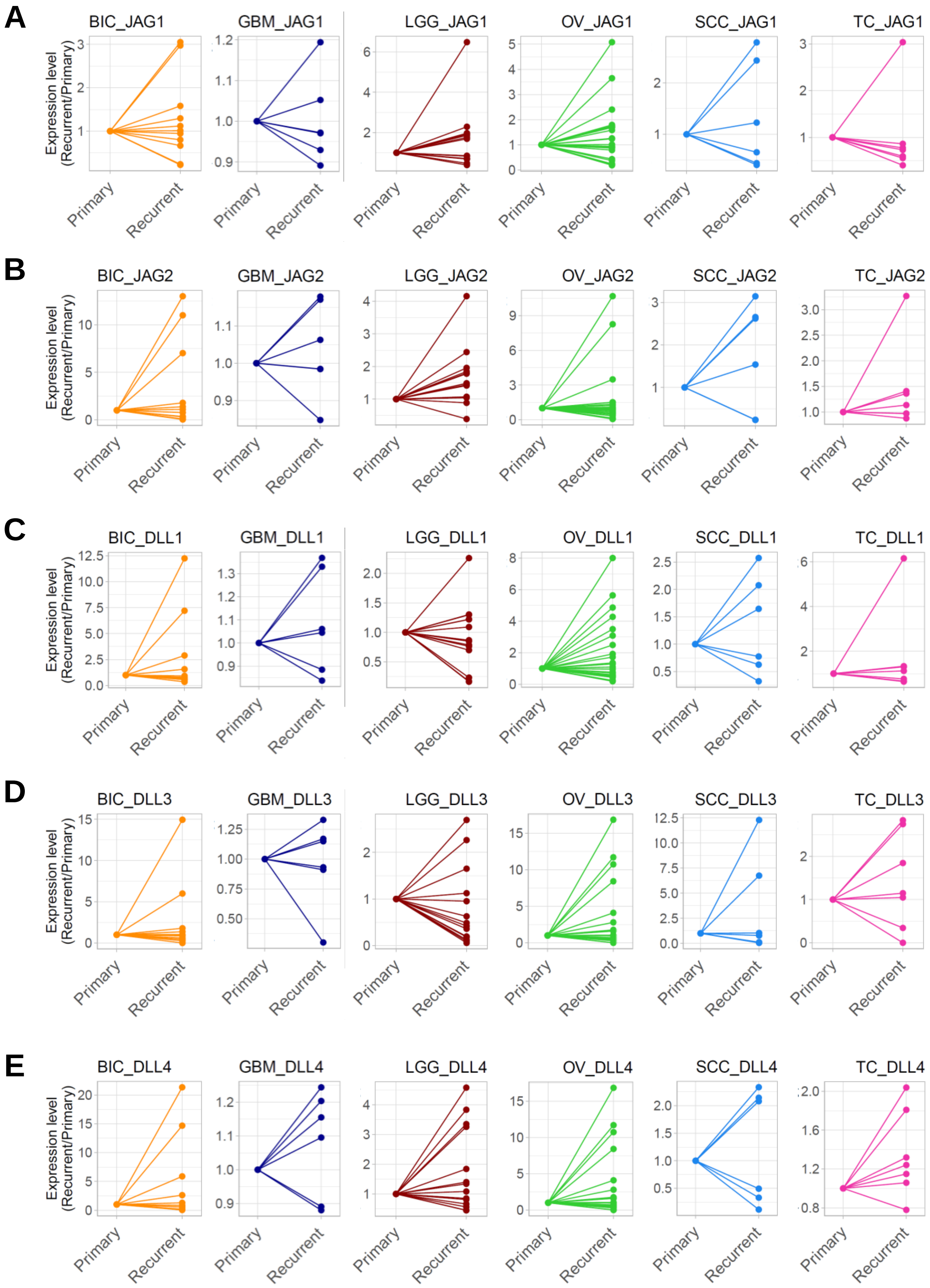

S13 Fig. Tracking mRNA expression of Notch ligands between primary and recurrent tumors of each patient with BIC, GBM, LGG, OV, SCC, and TC. Scatterplots for Notch ligands including (A) *JAG1*, (B) *JAG2*, (C) *DLL1*, (D) *DLL3*, and (E) *DLL4*.

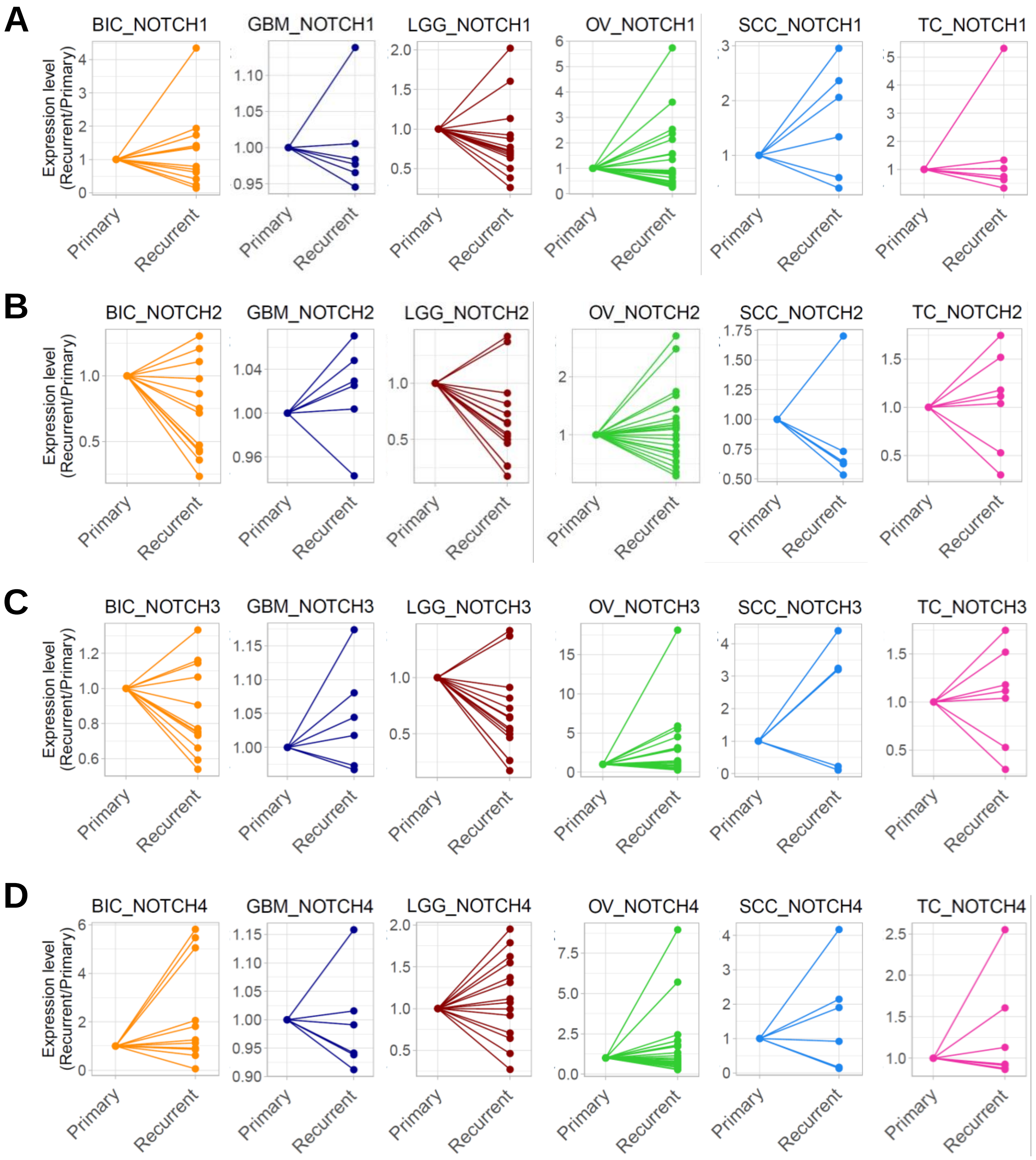

S14 Fig. Tracking mRNA expression of Notch receptors between primary and recurrent tumors of each patient with BIC, GBM, LGG, OV, SCC, and TC. Scatterplots for Notch receptors including (A) *NOTCH1*, (B) *NOTCH2*, (C) *NOTCH3*, and (D) *NOTCH4*.

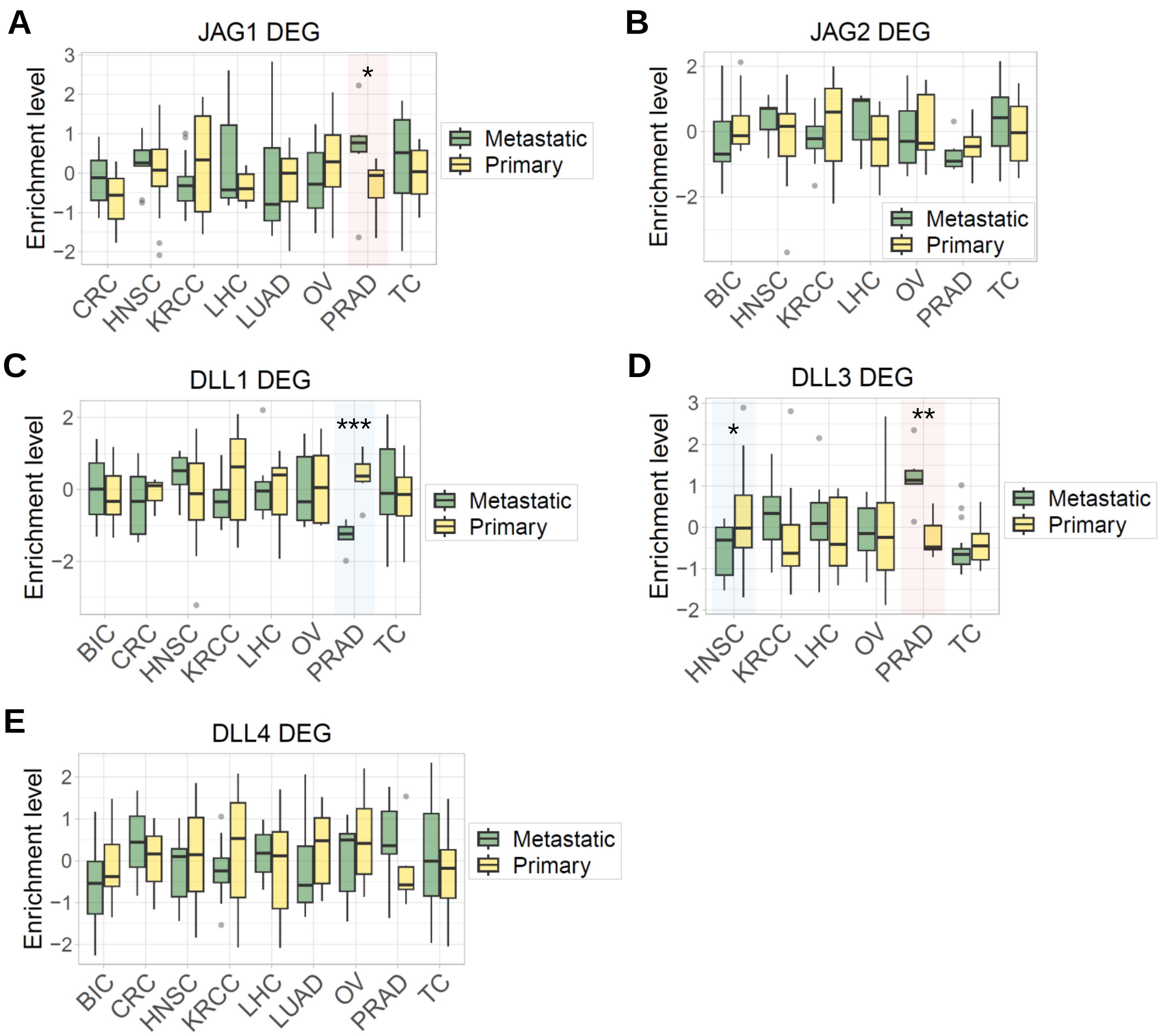

S15 Fig. Box plots showing the enrichment score of (A) JAG1 DEG, (B) JAG2 DEG, (C) DLL1 DEG, (D) DLL3 DEG, and (E) DLL4 DEG in primary and metastatic tumors. Data were analyzed using a two-tailed Student's t-test (*$p < 0.05$, **$p < 0.01$, ***$p < 0.001$).

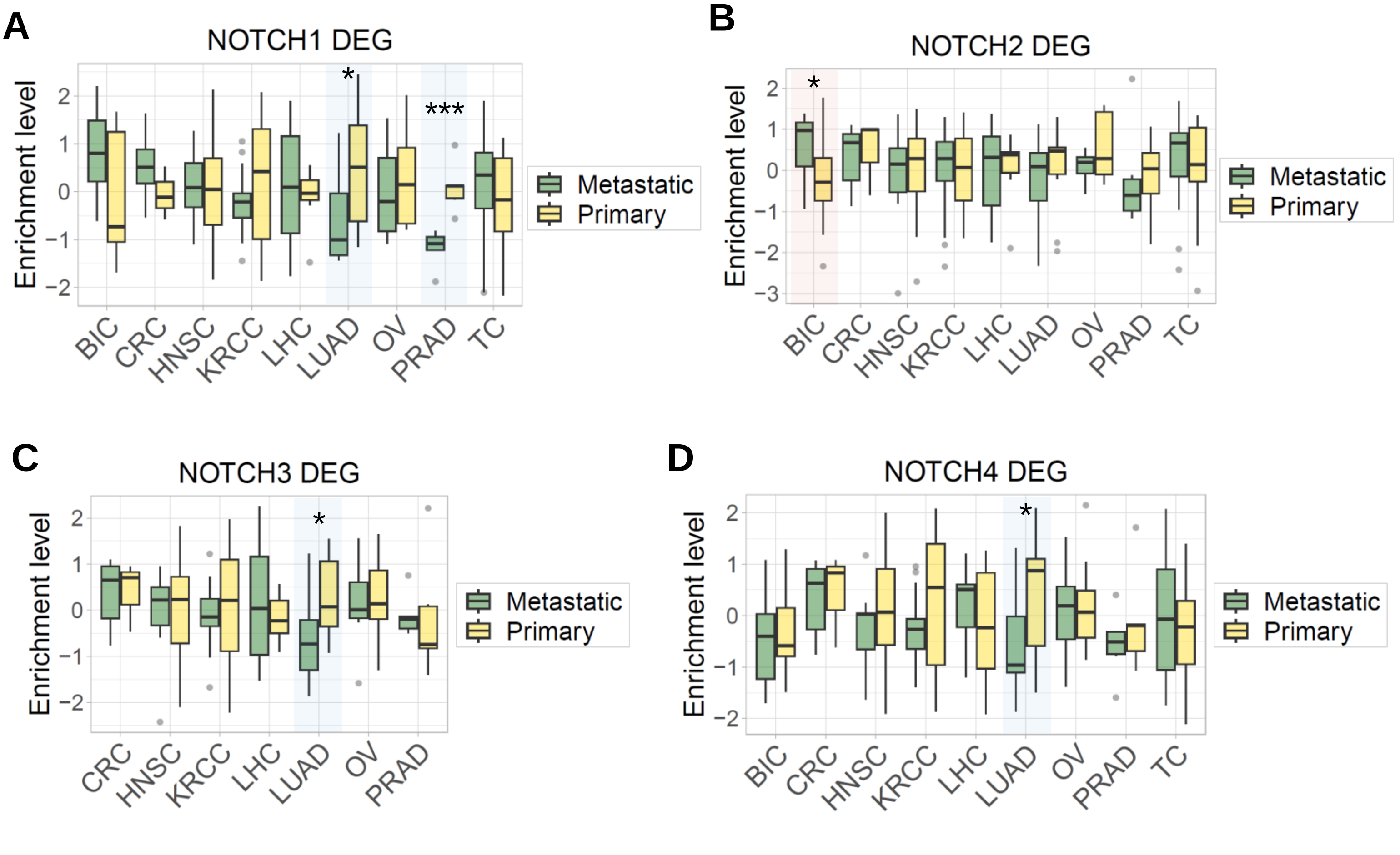

S16 Fig. Box plots showing the enrichment score of (A) NOTCH1 DEG, (B) NOTCH2 DEG, (C) NOTCH3 DEG, and (D) NOTCH4 DEG in primary and metastatic tumors. Data were analyzed using a two-tailed Student's t-test (*$p < 0.05$, ***$p < 0.001$).

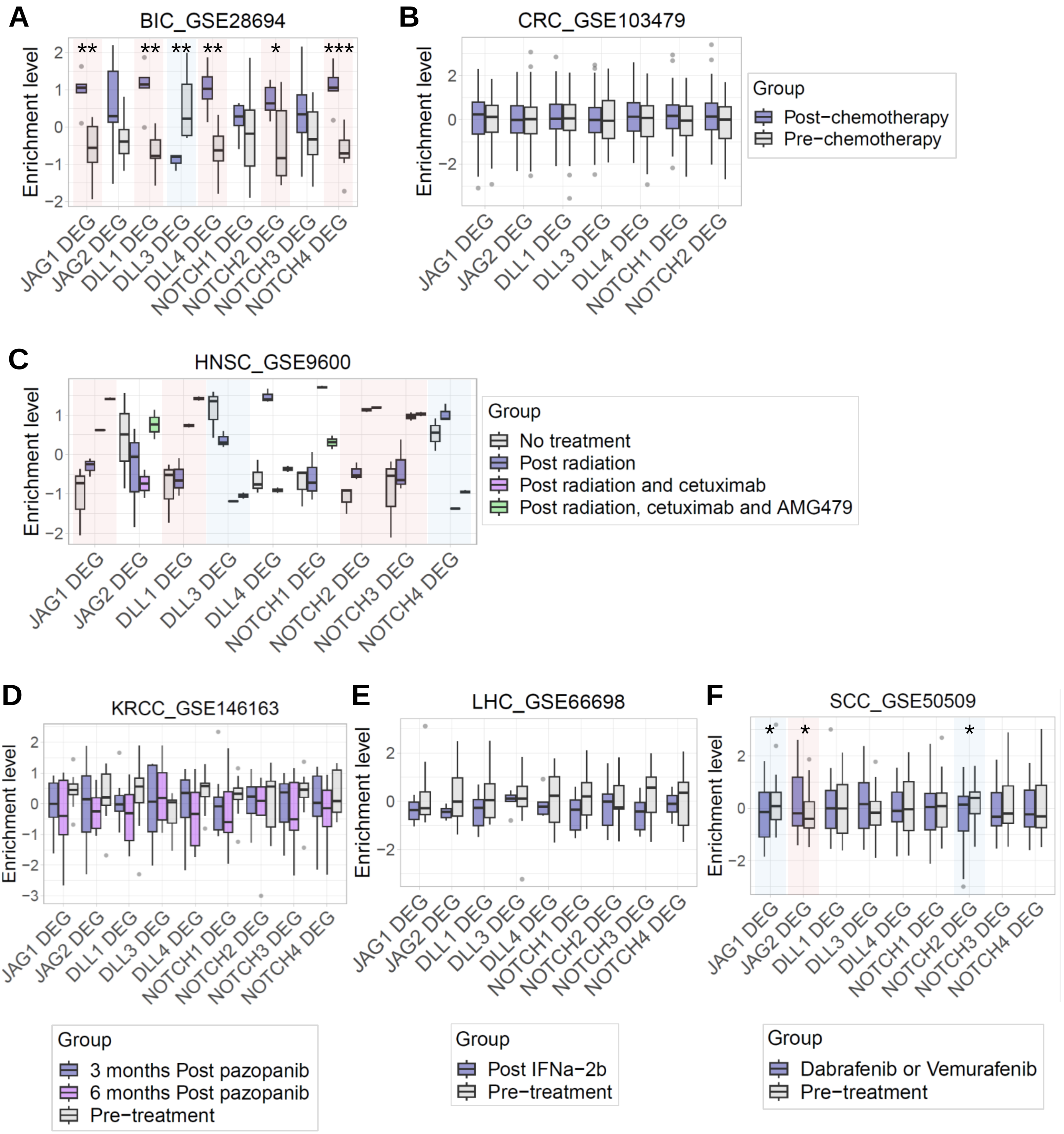

S17 Fig. Box plots comparing the enrichment score of DEG for each JAG, DLL, and NOTCH families in patients with anti-cancer drugs.

(A) Enrichment score of DEG for each JAG, DLL, and NOTCH families before and after chemotherapy in BIC. Data were analyzed using a two-tailed Student's t-test (*$p < 0.05$, ***$p < 0.001$).

(B) Enrichment score of DEG for each JAG, DLL, and NOTCH families before and after chemotherapy in CRC.

(C) Enrichment score of DEG for each of JAG, DLL, and NOTCH families before and after radiotherapy and chemotherapy with cetuximab and AMG479 in HNSC.

(D) Enrichment score of DEG for each JAG, DLL, and NOTCH families before, 3 months, and 6 months after chemotherapy with pazopanib in KRCC.

(E) Enrichment score of DEG for each JAG, DLL, and NOTCH families before and after chemotherapy with IFNa-2b in LHC.

(F) Enrichment score of DEG for each JAG, DLL, and NOTCH families before and after chemotherapy with dabrafenib or vemurafenib in SCC. Data were analyzed using a two-tailed Student's t-test.(*$p < 0.05$).

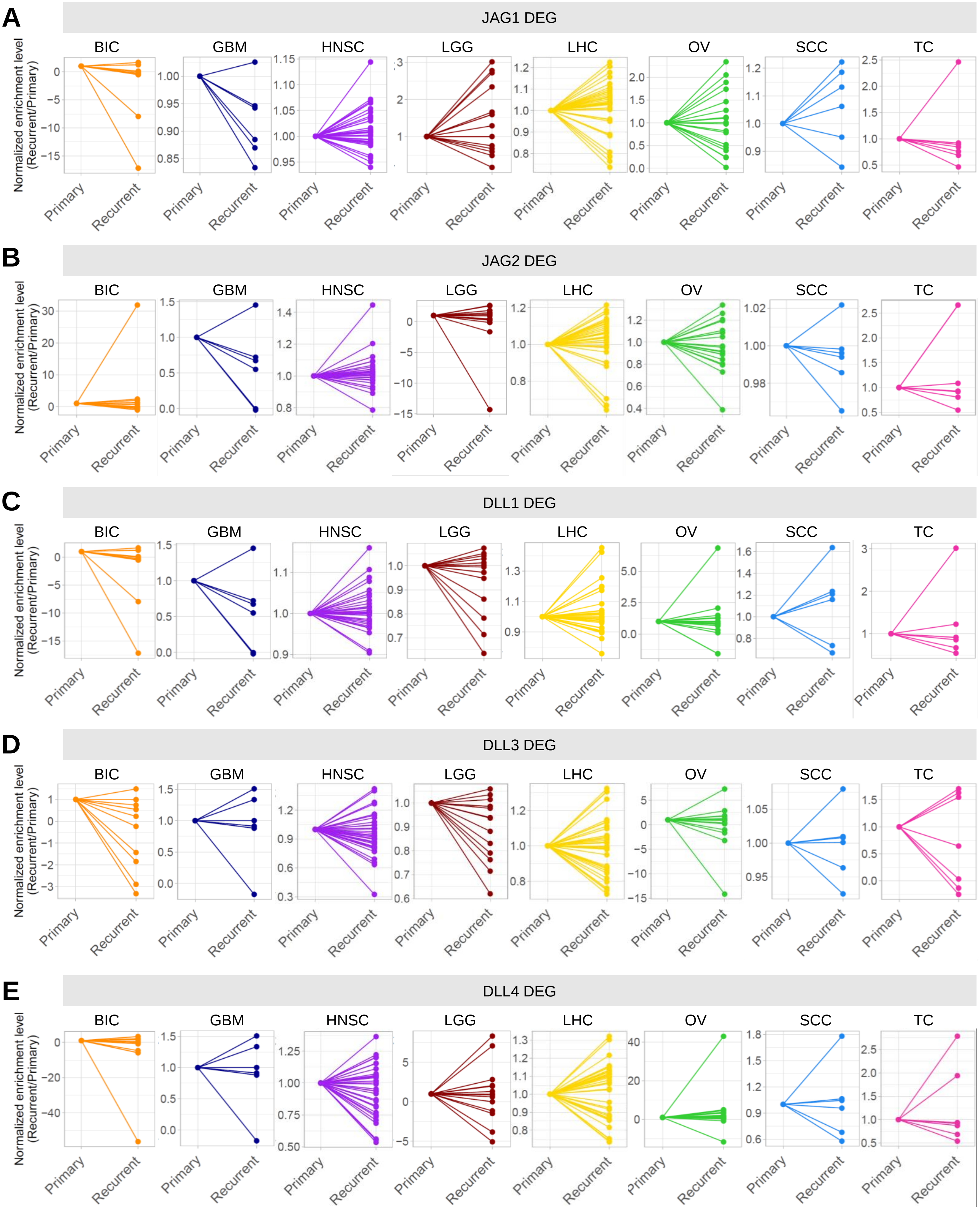

S18 Fig. Tracking enrichment score of DEG for each Notch ligand between primary and recurrent tumors of each patient with BIC, GBM, HNSC, LGG, LHC, OV, SCC, and TC. Scatterplots including (A) JAG1 DEG, (B) JAG2 DEG, (C) DLL1 DEG, (D) DLL3 DEG, and (E) DLL4 DEG.

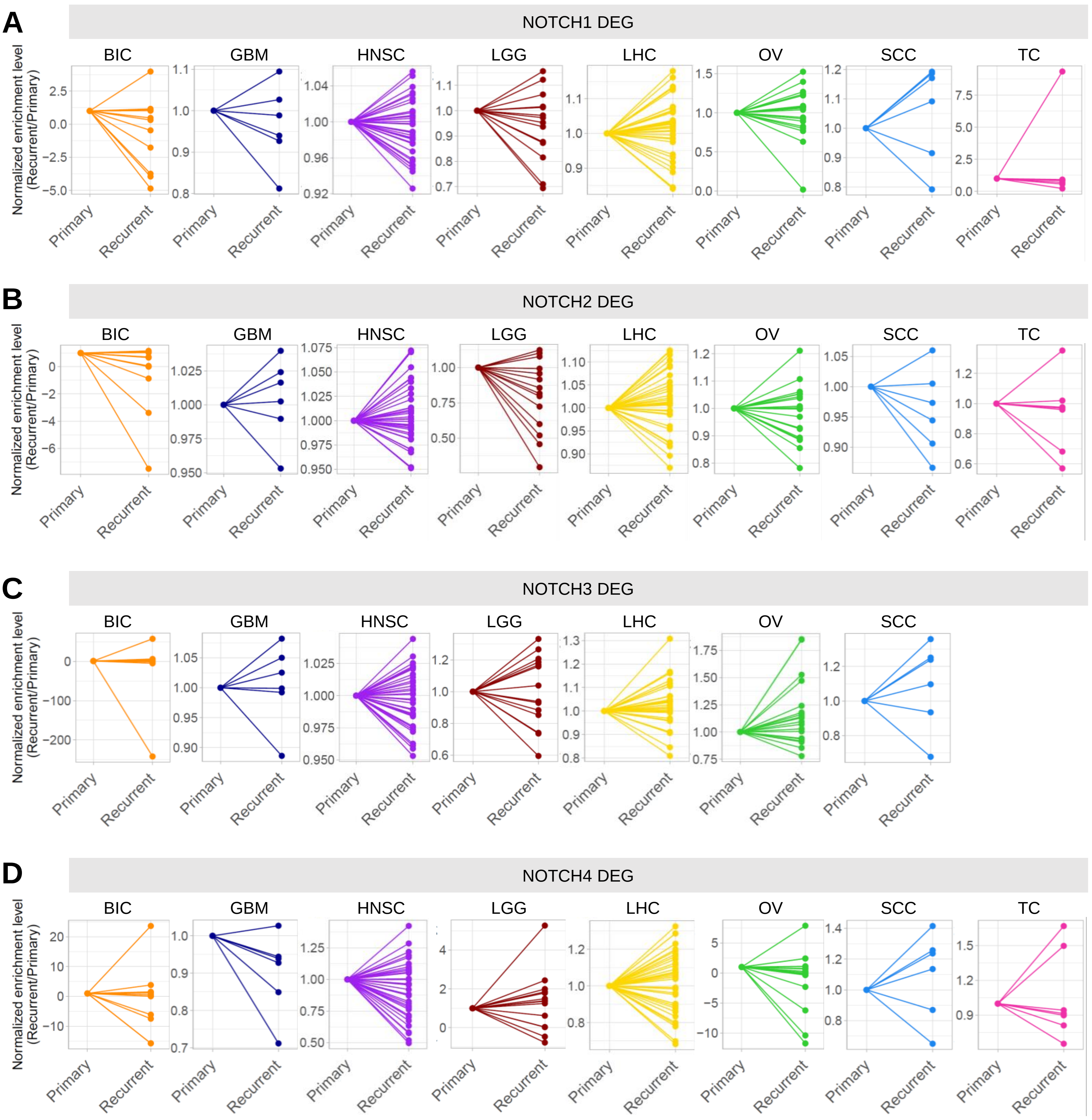

S19 Fig. Tracking enrichment score of DEG for each Notch receptor between primary and recurrent tumors of each patient with BIC, GBM, HNSC, LGG, LHC, OV, SCC, and TC. Scatterplots including (A) NOTCH1 DEG, (B) NOTCH2 DEG, (C) NOTCH3 DEG, and (D) NOTCH4 DEG.

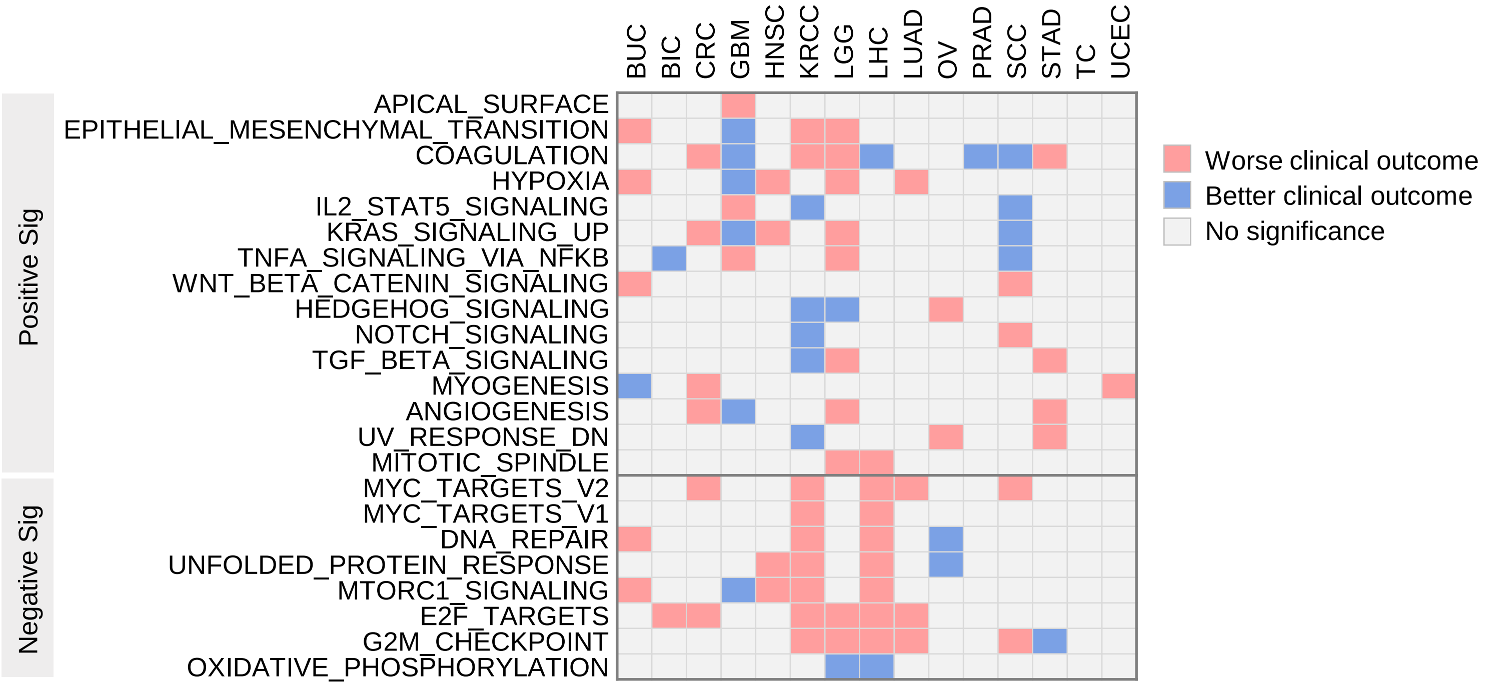

S20 Fig. A matrix showing the overall result from the survival analysis using the positive and negative signatures. The difference in survival, of which the *p* value from the log-rank t-test is less than 0.05, was presented to cause worse or better outcome.

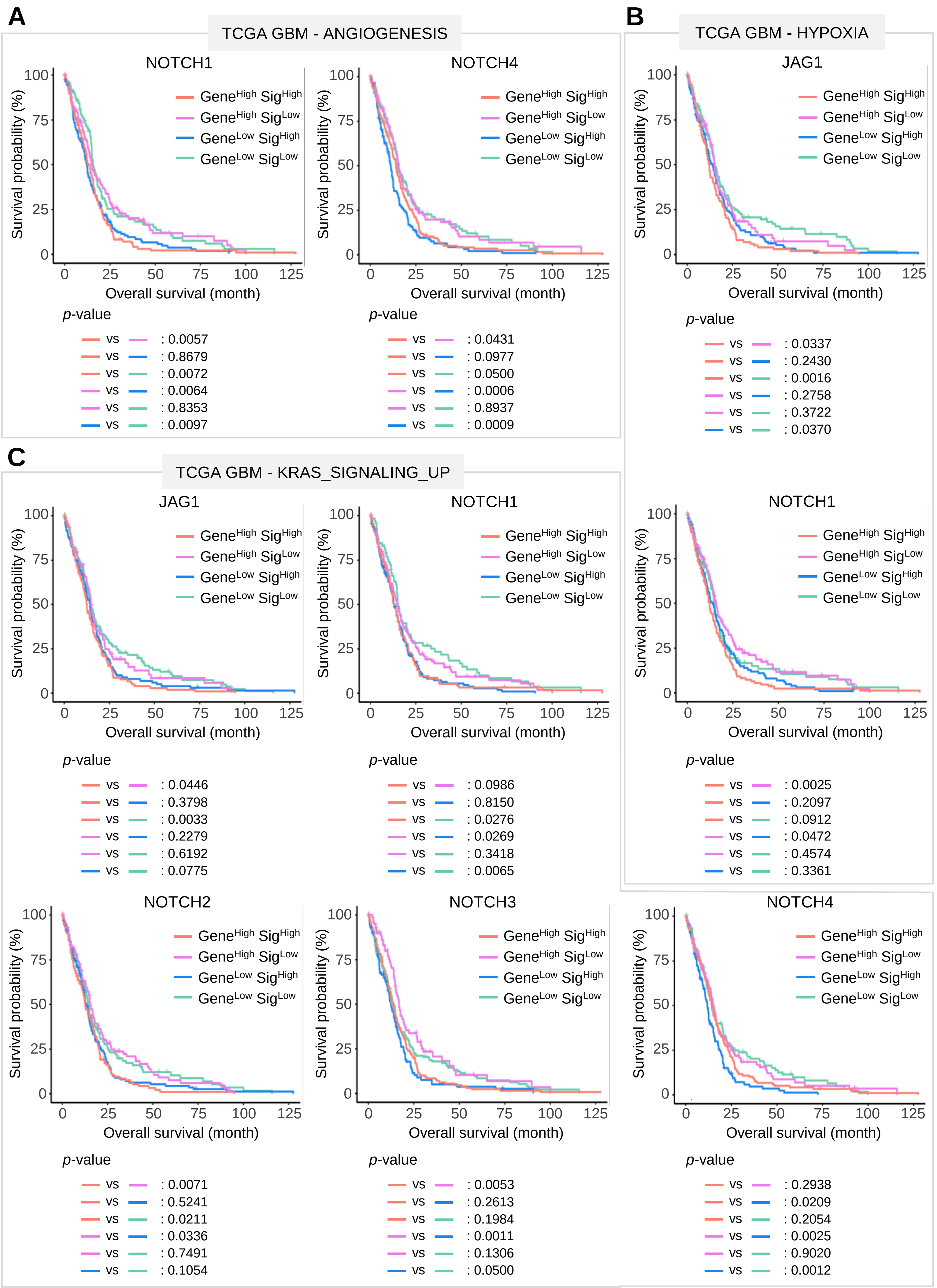

A
TCGA GBM - ANGIOGENESIS
NOTCH1
NOTCH4
B
TCGA GBM - HYPOXIA
JAG1
NOTCH1
C
TCGA GBM - KRAS_SIGNALING_UP
JAG1
NOTCH1
NOTCH2
NOTCH3
NOTCH4
NOTCH4
Survival probability (%)
Overall survival (month)
Gene^{High} Sig^{High}
Gene^{High} Sig^{Low}
Gene^{Low} Sig^{High}
Gene^{Low} Sig^{Low}
p-value
A NOTCH1: vs : 0.0057; vs : 0.8679; vs : 0.0072; vs : 0.0064; vs : 0.8353; vs : 0.0097
A NOTCH4: vs : 0.0431; vs : 0.0977; vs : 0.0500; vs : 0.0006; vs : 0.8937; vs : 0.0009
B JAG1: vs : 0.0337; vs : 0.2430; vs : 0.0016; vs : 0.2758; vs : 0.3722; vs : 0.0370
B NOTCH1: vs : 0.0025; vs : 0.2097; vs : 0.0912; vs : 0.0472; vs : 0.4574; vs : 0.3361
B NOTCH4: vs : 0.2938; vs : 0.0209; vs : 0.2054; vs : 0.0025; vs : 0.9020; vs : 0.0012
C JAG1: vs : 0.0446; vs : 0.3798; vs : 0.0033; vs : 0.2279; vs : 0.6192; vs : 0.0775
C NOTCH1: vs : 0.0986; vs : 0.8150; vs : 0.0276; vs : 0.0269; vs : 0.3418; vs : 0.0065
C NOTCH2: vs : 0.0071; vs : 0.5241; vs : 0.0211; vs : 0.0336; vs : 0.7491; vs : 0.1054
C NOTCH3: vs : 0.0053; vs : 0.2613; vs : 0.1984; vs : 0.0011; vs : 0.1306; vs : 0.0500

S21 Fig. Kaplan-Meier survival plots showing overall survival of GBM patients with respect to expression of JAG and NOTCH families, and enrichment of (A) Angiogenesis, (B) Hypoxia, and (C) KRAS signaling UP signatures.

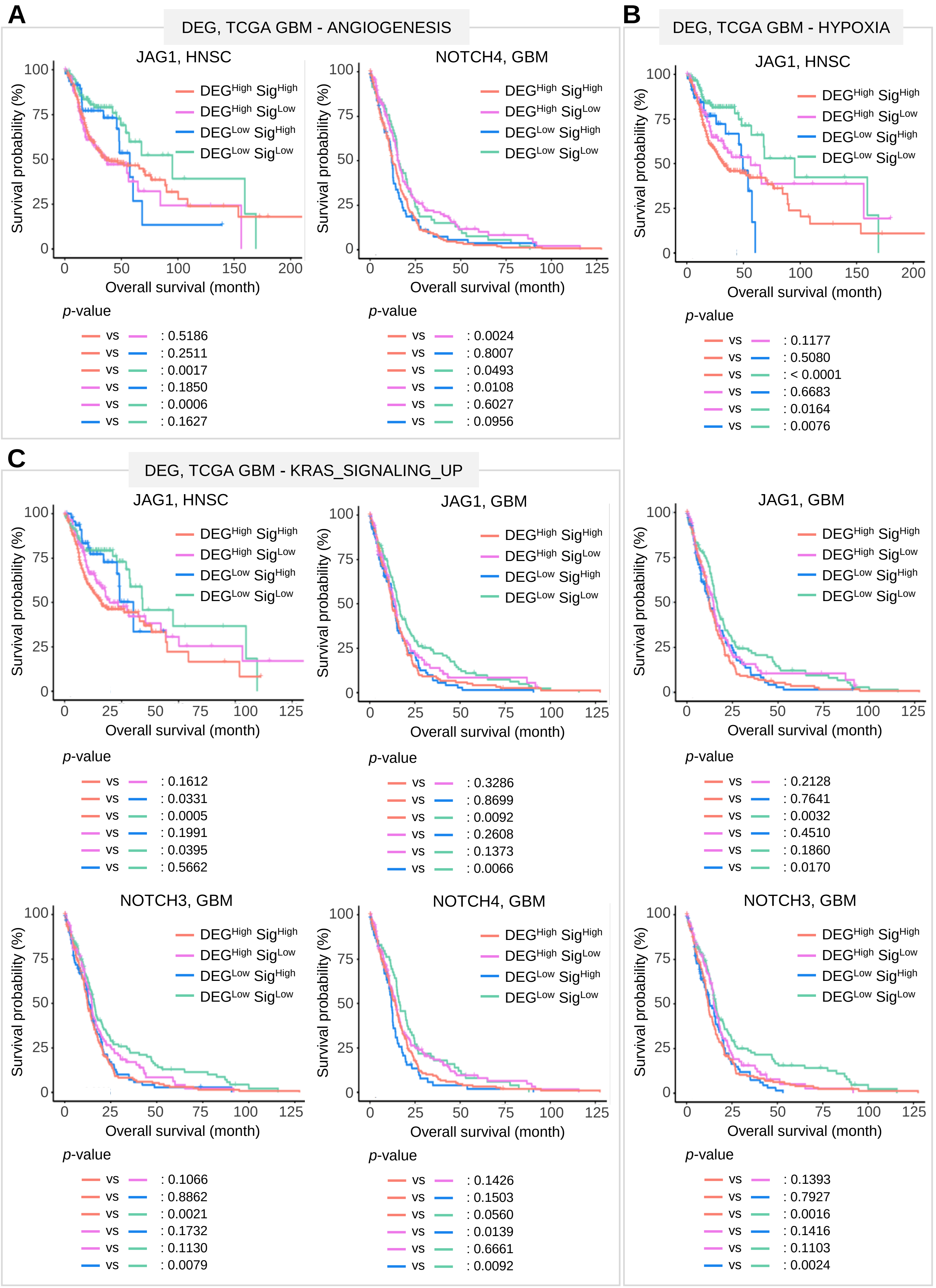
A
DEG, TCGA GBM - ANGIOGENESIS
JAG1, HNSC
Survival probability (%)
Overall survival (month)
DEG^High Sig^High
DEG^High Sig^Low
DEG^Low Sig^High
DEG^Low Sig^Low
p-value
vs : 0.5186
vs : 0.2511
vs : 0.0017
vs : 0.1850
vs : 0.0006
vs : 0.1627
NOTCH4, GBM
Survival probability (%)
Overall survival (month)
DEG^High Sig^High
DEG^High Sig^Low
DEG^Low Sig^High
DEG^Low Sig^Low
p-value
vs : 0.0024
vs : 0.8007
vs : 0.0493
vs : 0.0108
vs : 0.6027
vs : 0.0956
B
DEG, TCGA GBM - HYPOXIA
JAG1, HNSC
Survival probability (%)
Overall survival (month)
DEG^High Sig^High
DEG^High Sig^Low
DEG^Low Sig^High
DEG^Low Sig^Low
p-value
vs : 0.1177
vs : 0.5080
vs : < 0.0001
vs : 0.6683
vs : 0.0164
vs : 0.0076
JAG1, GBM
Survival probability (%)
Overall survival (month)
DEG^High Sig^High
DEG^High Sig^Low
DEG^Low Sig^High
DEG^Low Sig^Low
p-value
vs : 0.2128
vs : 0.7641
vs : 0.0032
vs : 0.4510
vs : 0.1860
vs : 0.0170
NOTCH3, GBM
Survival probability (%)
Overall survival (month)
DEG^High Sig^High
DEG^High Sig^Low
DEG^Low Sig^High
DEG^Low Sig^Low
p-value
vs : 0.1393
vs : 0.7927
vs : 0.0016
vs : 0.1416
vs : 0.1103
vs : 0.0024
C
DEG, TCGA GBM - KRAS_SIGNALING_UP
JAG1, HNSC
Survival probability (%)
Overall survival (month)
DEG^High Sig^High
DEG^High Sig^Low
DEG^Low Sig^High
DEG^Low Sig^Low
p-value
vs : 0.1612
vs : 0.0331
vs : 0.0005
vs : 0.1991
vs : 0.0395
vs : 0.5662
JAG1, GBM
Survival probability (%)
Overall survival (month)
DEG^High Sig^High
DEG^High Sig^Low
DEG^Low Sig^High
DEG^Low Sig^Low
p-value
vs : 0.3286
vs : 0.8699
vs : 0.0092
vs : 0.2608
vs : 0.1373
vs : 0.0066
NOTCH3, GBM
Survival probability (%)
Overall survival (month)
DEG^High Sig^High
DEG^High Sig^Low
DEG^Low Sig^High
DEG^Low Sig^Low
p-value
vs : 0.1066
vs : 0.8862
vs : 0.0021
vs : 0.1732
vs : 0.1130
vs : 0.0079
NOTCH4, GBM
Survival probability (%)
Overall survival (month)
DEG^High Sig^High
DEG^High Sig^Low
DEG^Low Sig^High
DEG^Low Sig^Low
p-value
vs : 0.1426
vs : 0.1503
vs : 0.0560
vs : 0.0139
vs : 0.6661
vs : 0.0092

S22 Fig. Kaplan-Meier survival plots demonstrating the prognostic outcomes of the patients with respect to the DEG enrichment of JAG, NOTCH families, and enrichment of (A) Angiogenesis, (B) Hypoxia, and (C) KRAS signaling UP signatures.

				Gene exp		DEG enrich	
Gene	Signature	Cancer type	Correlation	Sig High	Sig Low	Sig High	Sig Low
JAG1	Angiogenesis	KRCC	Positive	Better clinical outcome		Better clinical outcome	
	TGF beta signaling	KRCC	Positive			Better clinical outcome	
	DNA Repair	KRCC	Negative				Better clinical outcome
	Oxidative phosphorylation	PRAD	Negative				
JAG2	Angiogenesis	KRCC	Positive			Better clinical outcome	
	Apical junction	LHC	Negative				
	E2F targets	PRAD	Negative				
	Peroxisome	BUC	Negative				
DLL1	Hypoxia	PRAD	Positive				
	Apical junction	PRAD	Positive				
	E2F Targets	PRAD	Negative				
	Interferon alpha response	OV	Negative				
DLL3	DNA repair	PRAD	Positive				
	MYC Targets V1	LHC	Positive				
	KRAS signaling UP	TC	Negative				
	Apical junction	TC	Negative				
DLL4	Angiogenesis	HNSC	Positive				Better clinical outcome
	Angiogenesis	PRAD	Positive				
	E2F Targets	BIC	Negative	Worse clinical outcome			
	MYC Targets V1	BIC	Negative	Worse clinical outcome			
NOTCH1	Angiogenesis	KRCC	Positive	Better clinical outcome		Better clinical outcome	
	TGF beta signaling	KRCC	Positive			Better clinical outcome	
	DNA repair	LHC	Negative				
	DNA repair	PRAD	Negative				
NOTCH2	Angiogenesis	CRC	Positive				
	Apical junction	CRC	Positive		Worse clinical outcome		
	Oxidative phosphorylation	PRAD	Negative			Worse clinical outcome	
	DNA repair	PRAD	Negative				
NOTCH3	Angiogenesis	CRC	Positive	Worse clinical outcome		Worse clinical outcome	
	Apical junction	CRC	Positive	Worse clinical outcome	Worse clinical outcome		
	Fatty acid metabolism	OV	Negative				
	Adipogenesis	UCEC	Negative	Worse clinical outcome			
NOTCH4	Angiogenesis	CRC	Positive	Worse clinical outcome		Worse clinical outcome	
	Angiogenesis	STAD	Positive				Worse clinical outcome
	E2F Targets	BIC	Negative				
	MYC Targets V1	BIC	Negative				

Worse clinical outcome
Better clinical outcome
No significance

S23 Fig. Survival analysis using gene expression or DEGs along with the gene signatures correlated to JAG-NOTCH signaling pathway.
