# Supplementary material for: Cancer-wide in silico analyses using differentially expressed genes demonstrate the functions and clinical relevance of JAG, DLL, and NOTCH
Source: PLoS One. 2024 Jul 29;19(7):e0307943. doi: 10.1371/journal.pone.0307943 (PMC11285958; doi:10.1371/journal.pone.0307943)
Supplement: Attachment — Submitted filename: Response to Reviewers_Kim et al_PL.docx [file pone.0307943.s002.docx]

**A rebuttal letter for revision of PONE-D-23-37633**

**Cancer-wide in silico analyses using differentially expressed genes demonstrate the functions and clinical relevance of *JAG*, *DLL*, and *NOTCH***

Jung Yun Kim et al.

**Reviewer #1:**

1. Using in silico analysis, the investigator demonstrates the functions and clinical relevance of JAG, DLL, and NOTCH. But the data are only genetic data and survival data. There is a lack of information on other clinical and pathological characteristics of patients, which can not reflect the relationship between gene expression and disease in a panoramic way.

**Response:** Thank you for your insightful comment. To reflect the relationship between the gene expression of the JAG, DLL, and NOTCH families and clinical/pathological characteristics, we performed additional analyses. The analyses include comparisons between primary/metastatic, pre-/post-chemotherapy, and primary/recurrent tumor.

First, we investigated the gene expression of the JAG, DLL, and NOTCH families in primary or metastatic tumors (**Response Figure 1**). Changes in expression of the JAG and DLL families were diverse across each gene and cancer type. For example, *JAG2, DLL3,* and *DLL4* were increased in the metastatic tumors of PRAD, whereas *JAG1* decreased. In contrast, transcriptomic changes in the NOTCH family have only been observed in some cases.

Second, we compared expression between samples from patients before and after therapy (**Response Figure 2**). Although restricted to BIC and HNSC, the expression of NOTCH family did not change or decrease, whereas that of JAG and DLL increased (**Response Figure 2B and 2C**).

We also performed an analysis between primary and recurrent tumors in eight cases of tumor species (**Response Figure 3-5**). The samples were restricted to longitudinal biopsies from the same patient. In GBM, LGG, and HNSC, there were no differences in the expression of JAG, DLL, and NOTCH families between primary and recurrent tumors. In contrast, several patients with BIC, LHC, OV, SCC, and TC showed increased expression of JAG and DLL families without decline. However, the expression of NOTCH family in these patients showed diverse patterns without any tendency. Additionally, the expression of most of NOTCH family was slightly different, whereas that of JAG and DLL increased in several tumor types, such as CRC, HNSC, OV, PRAD, SCC, and TC.

Collectively, these results indicate that ligand-dependent NOTCH signaling regulation might play a predominant role among the various regulatory mechanisms involved in tumor progression, drug resistance, recurrence, and metastasis, consistent with findings from previous studies (1-3). In addition, receptor-independent activation of the JAG and DLL family may affect aggressiveness of tumor as previously reported (4-6).

**Response Figure 1 (Fig 5A and S10 Fig in revised manuscript)** Box plots comparing the expression of JAG, DLL, and NOTCH families in primary and metastatic tumor. Data were analyzed using a two-tailed student’s t-test (**p* < 0.05, ***p* < 0.01, ****p* < 0.001)

**Response Figure 2 (Fig 5B, 5C, and S11A-D Fig in revised manuscript) A** Box plots comparing the expression of JAG, DLL, and NOTCH families in before and after chemotherapy with IFNa-2b in LHC. Data were analyzed using a two-tailed student’s t-test (**p* < 0.05; ***p* < 0.01). **B** Box plots comparing the expression of JAG, DLL, and NOTCH families in before and after radiotherapy and chemotherapy with cetuximab and AMG479 in HNSC. Data were analyzed using a two-tailed student’s t-test (**p* < 0.05; ***p* < 0.01; ****p* < 0.001). **C** The expression of JAG, DLL, and NOTCH families in before and after chemotherapy in BIC. Data were analyzed using a two-tailed student’s t-test (**p* < 0.05; ****p* < 0.001). **D** The expression of JAG, DLL, and NOTCH families in before and after chemotherapy in CRC. **E** The expression of JAG, DLL, and NOTCH families in before, 3 months and 6 months after chemotherapy with pazopanib in KRCC. Data were analyzed using a two-tailed student’s t-test (**p* < 0.05). **F** The expression of JAG, DLL, and NOTCH families in before and after chemotherapy with dabrafenib or vemurafenib in SCC.

**Response Figure 3 (Fig 5D and 5E in revised manuscript)** Scatter plots showing the expression of JAG, DLL, and NOTCH families between primary and recurrent cancers of each patient with (D) LHC and (E) HNSC.

**Response Figure 4 (S12 Fig in revised manuscript)** Scatterplots for Notch ligands including (A) *JAG1*, (B) *JAG2*, (C) *DLL1*, (D) *DLL3*, and (E) *DLL4*.

**Response Figure 5 (S13 Fig in revised manuscript)** Scatterplots for Notch receptors including (A) *NOTCH1*, (B) *NOTCH2*, (C) *NOTCH3*, and (D) *NOTCH4*.

**Lines 441-445 of the revised manuscript.** “While the gene expression of JAG, DLL, and NOTCH families themselves did not show a significant correlation with tumor stage (as shown in S3 Fig), a considerable number of hallmark signatures were enriched in patients with highly expressed JAG, DLL, and NOTCH families (Fig 1C and 3B). As the hallmarks of cancer promote tumor progression, we performed further analyses to link the transcriptomes of the JAG, DLL, and NOTCH families to tumor progression.”

**Lines 460-489 of the revised manuscript.** “We compared the expression of the JAG, DLL, and NOTCH families in primary and metastatic tumors (Fig 5A and S10 Fig). Expression changes in JAG and DLL families varied among genes and cancer types. For example, metastatic KRCC tumors decreased the expression of *JAG1* and *DLL4*. Metastatic PRAD tumors increased the expression of *JAG2*, *DLL3*, and *DLL4* but *JAG1* decreased. However, the expression of the NOTCH family between primary and metastatic tumors differed only in a few cases (S10B-S10E Fig).

Furthermore, we compared the expression between samples from patients before and after anti-cancer therapy (Fig 5B, 5C and S11 Fig). The expression of the JAG, DLL, and NOTCH families changed before and after anti-cancer therapy in some cancers. Although restricted to BIC and HNSC, the expression of most JAG and DLL families increased after anti-cancer therapy. Most genes whose expression changed upon therapy showed gradual changes in HNSC and KRCC as the treatment progressed (Fig 5C and S11C Fig).

Additionally, we analyzed the expression of JAG, DLL, and NOTCH families in primary and recurrent tumors from longitudinal biopsies of the same patient (Fig 5D, 5E, S12 and S13 Fig). Expression of the JAG and DLL families increased in most patients with recurrent BIC, LHC, OV, SC, and TC. However, the expression of NOTCH family expression in these patients showed diverse patterns without any tendency. There were no differences in the expression of JAG, DLL, and NOTCH families between primary and recurrent tumors in GBM, LGG, and HNSC.

Our findings align with prior research on the correlation between the expression of JAG, DLL, and NOTCH families and clinical stages. For instance, DLL3 is more highly expressed in metastatic CRC tumors than in primary lesions, and its diagnostic role with prognostic value and therapeutic potential for anti-cancer therapy has been suggested (33). Similarly, DLL4 has been linked to invasion and metastasis, with its expression in TC holding prognostic significance (34). Moreover, the expression of JAG1 and DLL4 correlates with time to relapse in LHC (35).

Collectively, these findings suggest that Notch signaling may serve as a predominant regulatory mechanism throughout tumor progression, metastasis, resistance to therapy, and recurrence corroborating previous studies (36, 37). Additionally, the receptor-independent activation of the JAG and DLL families may influence tumor aggressiveness (29, 38, 39). In addition, our results hint at a previously unexplored relationship between Notch signaling, therapeutic effects, and the potential for recurrence.”

2.The study included as many as 15 cases of tumor species, and the expression of these genes was different in different tumor species and different clinical stages, and their functions were inconsistent, which could not give readers a clear message, express a clear idea, and lack of reference value for follow-up studies.

**Response:** Thank you for your constructive comment. Previously, comprehensive analyses of JAG, DLL, and NOTCH expression and their features associated with prognostic values, such as correlation with patient survival and biological pathways, were performed for individual tumor types (7-10). However, each result was analyzed under different analytic conditions. Thus, we focused on analyzing these genes under identical conditions and suggesting common or distinctive features of the JAG, DLL, and NOTCH families based on 50 cancer-hallmark gene sets.

Additionally, we investigated whether the expression levels of the JAG, DLL, and NOTCH families increased with tumor progression reflected by tumor stages (**Response Figure 6**). Stage-dependent upregulation of expression was observed for several genes including *JAG1* in GBM, LGG, and SCC. In contrast, in UCEC, the expression of *JAG1* decreased with tumor progression. Similarly, each gene exhibited a different trend in transcriptomic changes for each cancer type. However, there are limited cases showing tumor stage-dependent changes and simultaneously higher expression than in normal tissue.

Analyses of gene expression patterns according to clinical stages, such as pre-/post-therapy, primary/recurrent, and primary/metastatic tumors, demonstrated that the expression of JAG, DLL, and NOTCH families have diverse trends in each tumor and clinicopathological classification.

For example, *DLL1* was upregulated in metastatic and post-therapy tumors compared to that in primary and pre-therapy tumors, whereas there was no difference after recurrence in HNSC (**Response Figure 7A and 7B**). These results indicate that *DLL1* may be correlated with tumor metastasis and drug resistance, suggesting that DLL1 is a potential therapeutic target. In addition, although the expression of *DLL1* was not correlated with patient survival, the DLL1-DEGs enrichment was significantly correlated with a worse prognosis (**Response Figure 7C**). Furthermore, patient outcome was correlated with the enrichment of hallmark signatures, including hypoxia, KRAS signaling, unfolded protein response, and mTORC1 signaling (**Response Figure 7D**).

Moreover, the expression of JAG and NOTCH families was associated with patient prognosis, showing an enriched angiogenesis signature in GBM. The finding corresponds to responsiveness to combinatorial bevacizumab therapy with standard therapy (**Response Figure 8**).

Overall, while establishing a direct correlation between the stage-dependent expression of JAG, DLL, and NOTCH families and tumor progression remains challenging, our findings provide insight into predicting therapeutic response and the likelihood of recurrence in targeted therapy against Notch signaling.

**Response Figure 6 (S3 Fig in revised manuscript)** Box plots showing the gene expressions of (A) *JAG1*, (B) *JAG2*, (C) *DLL1*, (D) *DLL3*, (E) *DLL4*, (F) *NOTCH1*, (G) *NOTCH2*, (H) *NOTCH3*, and (I) *NOTCH4* according to tumor stage.

**Response Figure 7 (Fig 5A, 5C, 6A, and S14 Fig in revised manuscript) A** Box plots comparing the expression of DLL1 in primary and metastatic tumor. Data were analyzed using a two-tailed student’s t-test (**p* < 0.05; ***p* < 0.01; ****p* < 0.001). **B** Box plots comparing the expression of JAG, DLL, and NOTCH families in before and after radiotherapy and chemotherapy with cetuximab and AMG479 in HNSC. Data were analyzed using a two-tailed student’s t-test (**p* < 0.05; ***p* < 0.01; ****p* < 0.001). **C** A matrix demonstrating the overall result from the survival analysis using the expression levels or DEG enrichment scores of JAG, DLL, and NOTCH families in the datasets of each cancer type. **D** A matrix showing the overall result from the survival analysis using the positive and negative signatures. The difference in survival, of which the *p* value from the log-rank t-test is less than 0.05, was presented to cause worse or better outcome.

**Response Figure 8 (Fig 6B-D in revised manuscript) A** Kaplan-Meier survival plots showing overall survival of patients with GBM with respect to expression of *JAG1* or *NOTCH3* and enrichment of angiogenesis signature. **B** Kaplan-Meier survival plots demonstrating the prognostic outcomes of the patients with respect to the DEG enrichment of JAG1 and NOTCH3 and enrichment of angiogenesis signature. **C** Kaplan-Meier survival plots comparing the overall survival of the patients with GBM treated with bevacizumab in combination with conventional anti-cancer therapy by the expression of *JAG1*, *NOTCH2*, and *NOTCH3*.

**Lines 240-246 of the revised manuscript.** “To validate the correlation between the expression of JAG, DLL, and NOTCH families and tumor progress, we compared the expression according to the tumor stage (S3 Fig). Each gene showed a tendency that is different from the tumor stage in each cancer type. For example, *JAG1* increased in a stage dependent manner in GBM&LGG and SCC and decreased in a stage dependent manner in UCEC (S3A Fig). Most of JAG, DLL, and NOTCH families were stage dependently expressed in GBM (S3 Fig). However, there are limited cases showing tumor stage-dependent changes and having higher expression than normal tissue simultaneously.”

**Lines 460-463 of the revised manuscript.** “We compared the expression of the JAG, DLL, and NOTCH families in primary and metastatic tumors (Fig 5A and S10 Fig). Expression changes in JAG and DLL families varied among genes and cancer types. For example, metastatic KRCC tumors decreased the expression of *JAG1* and *DLL4*. Metastatic PRAD tumors increased the expression of *JAG2*, *DLL3*, and *DLL4* but *JAG1* decreased.”

**Lines 469-471 of the revised manuscript.** “Most genes whose expression changed upon therapy showed gradual changes in HNSC and KRCC as the treatment progressed (Fig 5C and S11C Fig).”

**Lines 516-522 of the revised manuscript.** “Survival analysis using expression and DEG enrichment demonstrated distinguishable results; for example, in KRCC, only *JAG1* expression led to better outcomes in patients with KRCC; however, the analysis with DEG enrichment showed that all the DEGs led to longer survival, except for the DLL3-DEGs (Fig 6A). In addition, *JAG1* expression did not affect clinical outcomes, whereas the enrichment of DEGs showed a negative impact on survival in GBM (Fig 6A). Similarly, survival analysis using the enrichment scores of the positive and negative signatures revealed the effect of the signatures on patient survival (S14 Fig).”

**Lines 530-540 of the revised manuscript.** “Survival analysis using the expression of JAG and NOTCH families and angiogenesis signature enrichment in GBM demonstrated that in the group expressing higher levels of these genes, angiogenesis signature enrichment negatively impacted patient survival (Fig 6B and S15A Fig). In addition, the results of the survival analysis using the DEGs and signature enrichment elucidated the relationships between JAG and NOTCH families, hypoxia, and KRAS signaling (Fig 6C and S15-S17 Fig). DEG enrichment in the JAG and NOTCH families was also associated with a similar patient prognosis to that analyzed with gene expression (Fig 6C and S16A Fig). We compared these results to the outcomes from a clinical study providing information regarding the survival of patients with GBM who received bevacizumab combined with standard therapy and the gene expression profiles of the tumor before treatment (Fig 6D). Survival analysis revealed that patients with higher expression of *JAG1* or *NOTCH2* or *NOTCH3* did not respond to therapy (Fig 6D).”

3.It is suggested that on the basis of this study, we should focus on one or several tumor species for more in-depth analysis and obtain more **clear** conclusions.

**Response:** Thank you for your suggestion. We conducted an analysis to assess the correlations between the hallmark signatures selected with positive or negative sum or *r* and the expression levels or DEG enrichment scores of the JAG, DLL, and NOTCH families. The results showed that the expression of JAG, DLL, and NOTCH family genes, except *DLL3*, was positively correlated with positive signatures and *vice versa* in most cancer types (**Response Figure 9**). Also, we investigated the global network involving the JAG, DLL, and NOTCH families and their DEGs which modulate various biological functions (**Response Figure 10**). Gene network analysis revealed that JAG, DLL, and NOTCH families were associated with genes regulating various signatures including Notch signaling, angiogenesis, hypoxia, and KRAS signaling. Collectively, these findings suggest an intricate interplay among the JAG, DLL, and NOTCH families, enabling them to collectively orchestrate common features across diverse tumor types.

Furthermore, patient survival analyses were conducted based on the gene expression levels of the JAG, DLL, and NOTCH families, DEG enrichment, and signature enrichment (**Response Figure 11**). Survival analysis based on gene expression, DEG enrichment scores, and signature enrichment individually showed different trends in accordance with each gene or cancer type.

We also compared patient outcomes according to the combination of gene expression or DEG enrichment with signature enrichment. The correlation with patient survival varied according to the combination and tumor type. For example, in GBM, patients with higher expression of JAG and NOTCH families or higher enrichment of DEGs with enriched angiogenesis signature showed a worse prognosis (**Response Figure 12 and 13**). These results were reflected in the responsiveness to combination therapy of bevacizumab with standard therapy in patients with GBM, according to the expression levels of *JAG1, NOTCH2,* and *NOTCH3*.

In KRCC, patients with higher enrichment of JAG1- or NOTCH1-DEGs combined with an enriched TGFβ signaling signature showed a better prognosis (**Response Figure 14**). Additionally, higher JAG1-DEGs enrichment with a lower DNA repair signature led to better clinical outcomes (**Response Figure 14**). Additionally, according to the combinations of gene expression or DEG enrichment with signature enrichment such as *DLL4* with E2F target in BIC and gene expression or DEGs of NOTCH2 and NOTCH3 with apical junction in CRC, patient survival varied among different cancer types, suggesting an independent function for each gene in certain cancer type (**Response Figure 14**).

Taken together, our results suggest that JAG, DLL, and NOTCH families genes interact with each other to modulate various biological features in a complex network and regulate specific features separately. Furthermore, through the analysis of correlations between the transcriptome, hallmark signature, and clinical relevance in patient cohorts, our results provide valuable insights for identifying specific phenotypes as therapeutic targets and predicting the therapeutic effects of specific drugs in individual patient.

**Response Figure 9 (Fig 3B in revised manuscript)** A heatmap showing the correlation between the hallmark signatures and expression levels (top) or DEG enrichment scores (bottom) of JAG, DLL, and NOTCH families.

**Response Figure 10 (Fig S6 in revised manuscript)** Gene network plots for each signature including (A) Notch signaling, (B) Angiogenesis, (C) Hypoxia, and (D) KRAS signaling UP. Black dots indicate the genes belonging to the DEGs, which are involved in the regulatory network of the signature. Grey dots denote the mediator which are not included in the DEGs but essential for the regulation of the signature. (E) A table showing the compositions of each interaction category described in the gene network analysis.

**Response Figure 11 (Fig 6A and S14 Fig in revised manuscript) A** A matrix demonstrating the overall result from the survival analysis using the expression levels or DEG enrichment scores of JAG, DLL, and NOTCH families in the datasets of each cancer type. **B** A matrix showing the overall result from the survival analysis using the positive and negative signatures. The difference in survival, of which the *p* value from the log-rank t-test is less than 0.05, was presented to cause worse or better outcome.

**Response Figure 12 (Fig. 6B-D in revised manuscript) A** Kaplan-Meier survival plots showing overall survival of patients with GBM with respect to expression of JAG1 or NOTCH3 and enrichment of angiogenesis signature. **B** Kaplan-Meier survival plots demonstrating the prognostic outcomes of the patients with respect to the DEG enrichment of JAG1 and NOTCH3 and enrichment of angiogenesis signature. **C** Kaplan-Meier survival plots comparing the overall survival of the patients with GBM treated with bevacizumab in combination with conventional anti-cancer therapy by the expression of *JAG1*, *NOTCH2*, and *NOTCH3*.

**Response Figure 13 (S15A and S16A Fig in revised manuscript) A** Kaplan-Meier survival plots showing overall survival of GBM patients with respect to expression of JAG and NOTCH families, and enrichment of Angiogenesis. **B** Kaplan-Meier survival plots demonstrating the prognostic outcomes of the patients with respect to the DEG enrichment of JAG, NOTCH families, and enrichment of Angiogenesis.

**Response Figure 14 (S17 Fig in revised manuscript)** Survival analysis using gene expression or DEGs along with the gene signatures correlated to JAG-NOTCH signaling pathway.

**Lines 361-364 of the revised manuscript.** “Positive signatures generally showed a positive correlation with the expression levels and DEG enrichment scores of the JAG, DLL, and NOTCH families, while negative signatures displayed a negative overall correlation, except for *DLL3* which showed an opposite trend (Fig 3B).”

**Lines 376-384 of the revised manuscript.** “We conducted gene network analysis using the Cytoscape GeneMANIA plugin with the DEGs selected in GBM to validate the interactions between JAG, DLL, and NOTCH families and other genes in regulating of each signature (S6 Fig). The analysis provided a map of the interactions between the DEGs by integrating information from previous studies. The types of interactions and relative weights of the networks are represented by the colors and thicknesses of the lines, respectively. The mediators that are not included in the DEGs but are essential for the interactions are marked with gray circles, the size of which denotes the interaction score with the DEGs. Gene network plots corresponding to signatures, including NOTCH signaling, angiogenesis, hypoxia, and KRAS signaling, are presented in S6 Fig.”

**Lines 516-522 of the revised manuscript.** “Survival analysis using expression and DEG enrichment demonstrated distinguishable results; for example, in KRCC, only *JAG1* expression led to better outcomes in patients with KRCC; however, the analysis with DEG enrichment showed that all the DEGs led to longer survival, except for the DLL3-DEGs (Fig 6A). In addition, *JAG1* expression did not affect clinical outcomes, whereas the enrichment of DEGs showed a negative impact on survival in GBM (Fig 6A). Similarly, survival analysis using the enrichment scores of the positive and negative signatures revealed the effect of the signatures on patient survival (S14 Fig).”

**Lines 530-540 of the revised manuscript.** “Survival analysis using the expression of JAG and NOTCH families and angiogenesis signature enrichment in GBM demonstrated that in the group expressing higher levels of these genes, angiogenesis signature enrichment negatively impacted patient survival (Fig 6B and S15A Fig). In addition, the results of the survival analysis using the DEGs and signature enrichment elucidated the relationships between JAG and NOTCH families, hypoxia, and KRAS signaling (Fig 6C and S15-S17 Fig). DEG enrichment in the JAG and NOTCH families was also associated with similar patient prognosis to that analyzed with gene expression (Fig 6C and S16A Fig). We compared these results to the outcomes from a clinical study providing information regarding the survival of patients with GBM who received bevacizumab combined with standard therapy and the gene expression profiles of the tumor before treatment (Fig 6D). Survival analysis revealed that patients with higher expression of *JAG1* or *NOTCH2* or *NOTCH3* did not respond to therapy (Fig 6D).”

4. Please provide clear pictures and diagrams as required by the journal.

**Response:** We apologize for the inconvenience caused by the figures that did not meet the journal’s criteria. We have revised all figures and diagrams, as well as adjusted the font size in accordance to the guidelines.

**Reviewer #2:** Title: Cancer-wide in silico analyses using differentially expressed genes demonstrate the functions and clinical relevance of JAG, DLL, and NOTCH

Major Comments:

A. Methods:

Data download and processing:

1. Justification for dataset selection: It would be helpful if the authors provided a brief rationale for selecting these specific datasets. This information would enhance the readers understanding of the dataset suitability and reinforce the relevance of the subsequent analyses. Furthermore, reason for using GlioVis data portal instead of TCGA GBM datasets for the study.

**Response:** Thank you for the suggestion. The Cancer Genome Atlas (TCGA) program provides transcriptomic data for primary cancers across various cancer types. Among multiple datasets, we selected the one including the largest number of patient samples for further analysis. The datasets, except GBM, were downloaded from cBioPortal as there was no normal tissue samples included in the identical dataset with primary tumor of GBM (11). For GBM, the dataset including both primary tumor and normal tissue samples, which was also from TCGA, was downloaded from the GlioVis data portal (12). To avoid any confusion, we revised former phrases into more precise languages.

**Lines 97-99 of the revised manuscript.** “We downloaded the TCGA datasets containing more than 400 samples from cBioPortal for Cancer Genomics, except for GBM. For GBM, the dataset including both primary tumor and normal tissue samples, which is also from TCGA, was downloaded from GlioVis data portal (20).”

2. Justification for not using Normal tissue datasets: It will give a clear picture if author analyses the normal tissue dataset which can help to distinguish better between cancer-specific effects.

**Response:** Thank you for your insightful comment. In the analyses of the expression of the JAG, DLL, and NOTCH families, a comparison between primary tumor and normal tissue samples from the corresponding organs included in the same dataset was performed (**Response Figure 15**). The results showed that the overall expression of the JAG, DLL, and NOTCH families in tumor samples compared to that in normal tissues was distinct for each cancer type (**Response Figure 15A**). For BIC and OV, the datasets from TCGA provided only primary tumor samples.

To provide a clear explanation for the cancer-specific effects, we conducted further analyses to compare the expression of JAG, DLL, and NOTCH families between primary tumors and normal tissues using additional datasets (13, 14). As a result, in BIC, *JAG2*, *DLL3*, *DLL4*, and *NOTCH4* were downregulated in tumors compared to normal tissues, whereas other genes showed no significant differences (**Response Figure 15B**). In contrast, *JAG2* and *NOTCH3* were upregulated compared to normal tissues, whereas downregulation was observed only in *DLL1* in OV (**Response Figure 15B**).

**Response Figure 15 (Fig 1B and S2A Fig in revised manuscript) A** Box plots comparing the gene expressions of JAG, DLL, and NOTCH families in each cancer type and the corresponding normal tissues. Data were analyzed using a two-tailed student’s t-test (**p* < 0.05; ***p* < 0.01; ****p* < 0.001). **B** Box plots comparing the gene expressions of JAG, DLL, and NOTCH families in BIC (GSE65194) and OV (GSE69428) and the corresponding normal tissues. Data were analyzed using a two-tailed student’s t-test (**p* < 0.05; ***p* < 0.01; ****p* < 0.001).

**Lines 101-105 of the revised manuscript.** “The comparison of primary tumor and normal tissue was performed based on each TCGA datasets which included the transcriptome of tumor sample and corresponding normal tissue. The datasets of BIC and OV from TCGA provided only tumor samples. To compare tumor and normal in BIC and OV, we obtained additional datasets with the accession numbers GSE65194 and GSE69428 curated by Gene Expression Omnibus (GEO).”

**Lines 222-225 of the revised manuscript.** “First, we compared the mRNA expression levels of the JAG, DLL, and NOTCH families in each cancer type and corresponding normal tissues (Fig 1B and S2A Fig). The results showed a diverse tendency of expression across cancer types; their expression was either higher or lower than that in normal tissues.”

3. Justification for not evaluating the effects on additional clinical outcomes like treatment response, metastasis, recurrence: Analysis of just single factor like overall survival is a limiting factor of this study and does decrease the generalizability of the study.

**Response:** Thank you for your comment. To investigate the relationship between the gene expression of JAG, DLL, and NOTCH family and clinicopathological characteristics, we performed additional analyses comparing primary/metastatic, pre-/post-chemotherapy samples, and primary/recurrent tumors.

First, we analyzed the gene expression of the JAG, DLL, and NOTCH families in primary and metastatic tumors (**Response Figure 16**). Expression changes in JAG and DLL families varied among genes and cancer types. For example, PRAD metastasis showed increased expression of *JAG2*, *DLL3*, and *DLL4* whereas that of *JAG1* decreased.

Next, we compared expression between samples from patients before and after therapy (**Response Figure 17**). Although restricted to BIC and HNSC, the expression of the JAG and DLL families increased, whereas that of the NOTCH family did not change or decrease. In addition, the expression levels of most of the NOTCH family were slightly different whereas those of JAG and DLL families increased in several tumor types, such as CRC, HNSC, OV, PRAD, SCC, and TC.

In addition, we performed an analysis between primary and recurrent tumors from longitudinal biopsies from identical patients with eight tumor species (**Response Figure 18-20**). There were no differences in the expression of JAG, DLL, and NOTCH families between primary and recurrent GBM, LGG, and HNSC tumors. Meanwhile, the expression of the JAG and DLL families increased in several patients with BIC, LHC, OV, SC, and TC, without a decline. However, the expression of NOTCH family in these patients showed diverse patterns.

In conclusion, these results suggest that ligand-dependent NOTCH signaling modulation may affect tumor progression, drug resistance, metastasis, and recurrence, which is in line with previous studies (1-3). Additionally, receptor-independent activation of the JAG and DLL families may affect tumor aggressiveness as previously reported (4-6).

**Response Figure 16 (Fig 5A and S10 Fig in revised manuscript)** Box plots comparing the expression of JAG, DLL, and NOTCH families in primary and metastatic tumor. Data were analyzed using a two-tailed student’s t-test (**p* < 0.05, ***p* < 0.01, ****p* < 0.001).

**Response Figure 17 (Fig 5B, 5C, and S11A-D Fig in revised manuscript) A** Box plots comparing the expression of JAG, DLL, and NOTCH families in before and after chemotherapy with IFNa-2b in LHC. Data were analyzed using a two-tailed student’s t-test (**p* < 0.05; ***p* < 0.01). **B** Box plots comparing the expression of JAG, DLL, and NOTCH families in before and after radiotherapy and chemotherapy with cetuximab and AMG479 in HNSC. Data were analyzed using a two-tailed student’s t-test (**p* < 0.05; ***p* < 0.01; ****p* < 0.001). **C** The expression of JAG, DLL, and NOTCH families in before and after chemotherapy in BIC. Data were analyzed using a two-tailed student’s t-test (**p* < 0.05; ****p* < 0.001). **D** The expression of JAG, DLL, and NOTCH families in before and after chemotherapy in CRC. **E** The expression of JAG, DLL, and NOTCH families in before, 3 months and 6 months after chemotherapy with pazopanib in KRCC. Data were analyzed using a two-tailed student’s t-test (**p* < 0.05). **F** The expression of JAG, DLL, and NOTCH families in before and after chemotherapy with dabrafenib or vemurafenib in SCC.

**Response Figure 18 (Fig 5D and 5E in revised manuscript)** Scatter plots showing the expression of JAG, DLL, and NOTCH families between primary and recurrent cancers of each patient with (D) LHC and (E) HNSC.

**Response Figure 19 (S12 Fig in revised manuscript)** Scatterplots for Notch ligands including (A) *JAG1*, (B) *JAG2*, (C) *DLL1*, (D) *DLL3*, and (E) *DLL4*.

**Response Figure 20 (S13 Fig in revised manuscript)** The scatterplots for Notch receptors including (A) *NOTCH1*, (B) *NOTCH2*, (C) *NOTCH3*, and (D) *NOTCH4*.

**Lines 441-445 of the revised manuscript.** “While the gene expression of JAG, DLL, and NOTCH families themselves did not show a significant correlation with tumor stage (as shown in S3 Fig), a considerable number of hallmark signatures were enriched in patients with highly expressed JAG, DLL, and NOTCH families (Fig 1C and 3B). As the hallmarks of cancer promote tumor progression, we performed further analyses to link the transcriptomes of the JAG, DLL, and NOTCH families to tumor progression.”

**Lines 460-489 of the revised manuscript.** “We compared the expression of the JAG, DLL, and NOTCH families in primary and metastatic tumors (Fig 5A and S10 Fig). Expression changes in JAG and DLL families varied among genes and cancer types. For example, metastatic KRCC tumors decreased the expression of *JAG1* and *DLL4*. Metastatic PRAD tumors increased the expression of *JAG2*, *DLL3*, and *DLL4* but JAG1 decreased. However, the expression of the NOTCH family between primary and metastatic tumors differed only in a few cases (S10B-S10E Fig).

Furthermore, we compared the expression between samples from patients before and after anti-cancer therapy (Fig 5B, 5C and S11 Fig). The expression of the JAG, DLL, and NOTCH families changed before and after anti-cancer therapy in some cancers. Although restricted to BIC and HNSC, the expression of most JAG and DLL families increased after anti-cancer therapy. Most genes whose expression changed upon therapy showed gradual changes in HNSC and KRCC as the treatment progressed (Fig 5C and S11C Fig).

Additionally, we analyzed the expression of JAG, DLL, and NOTCH families in primary and recurrent tumors from longitudinal biopsies of the same patient (Fig 5D, 5E, S12 and S13 Fig). Expression of the JAG and DLL families increased in most patients with recurrent BIC, LHC, OV, SC, and TC. However, the expression of NOTCH family expression in these patients showed diverse patterns without any tendency. There were no differences in the expression of JAG, DLL, and NOTCH families between primary and recurrent tumors in GBM, LGG, and HNSC.

Our findings align with prior research on the correlation between the expression of JAG, DLL, and NOTCH families and clinical stages. For instance, DLL3 is more highly expressed in metastatic CRC tumors than in primary lesions, and its diagnostic role with prognostic value and therapeutic potential for anti-cancer therapy has been suggested (33). Similarly, DLL4 has been linked to invasion and metastasis, with its expression in TC holding prognostic significance (34). Moreover, the expression of JAG1 and DLL4 correlates with time to relapse in LHC (35).

Collectively, these findings suggest that Notch signaling may serve as a predominant regulatory mechanism throughout tumor progression, metastasis, resistance to therapy, and recurrence corroborating previous studies (36, 37). Additionally, the receptor-independent activation of the JAG and DLL families may influence tumor aggressiveness (29, 38, 39). In addition, our results hint at a previously unexplored relationship between Notch signaling, therapeutic effects, and the potential for recurrence.”

4. Explanation on using only mRNA expression data: it would be helpful if author will incorporate proteomic and metabolic data for clear insights.

**Response:** Thank you for your insightful comment. As you have mentioned, incorporating proteomic data would provide a more accurate understanding of the role of the JAG, DLL, and NOTCH families in cancer owing to their molecular characteristics. The JAG, DLL, and NOTCH family of proteins undergo proteolytic cleavage, forming an intracellular domain that exhibits the transcriptional regulatory functions of target genes (15). These processes depend on ligand-receptor interactions in canonical pathways (1,2). However, in some cases, they are ligand-receptor interaction-independent (4-6).

Thus, we investigated the protein expression of the JAG, DLL, and NOTCH families comparing primary tumors and normal tissues (**Response Figure 17**). The analyses included 10 cases (BIC, CRC, GBM, HNSC, KRCC, LGG, LHC, LUAD, OV, and UCEC) among the 15 cancer types, with expression data for both primary tumors and normal tissues. The results demonstrated that the overall expression of the JAG, DLL, and NOTCH families in tumor samples compared to normal tissue was distinguishable in each cancer type. For example, *JAG1*, *NOTCH1*, *NOTCH2*, and *NOTCH3* were upregulated in GBM but downregulated in LUAD, except *NOTCH3*, which is upregulated. In addition, a comparison of primary tumors and normal tissues at the transcriptomic and proteomic levels showed different trends for each cancer type (**Response Figure 21 and 22**). For example, most JAG, DLL, and NOTCH family genes were upregulated in primary tumors of LUAD compared to normal tissues at both the transcriptomic and proteomic levels. In contrast, the transcriptome and proteome showed opposite expression patterns in GBM.

As JAG, DLL, and NOTCH family proteins undergo cleavage, translocate into the nucleus, and regulate the transcription of target genes, transcriptional activation might indicate the activation of the JAG, DLL, and NOTCH families (5, 16). Thus, we performed patient survival analysis and demonstrated that DEG enrichment of the JAG, DLL, and NOTCH families was more closely correlated with patient prognosis than gene expression alone (**Response Figure 17**).

Collectively, the mRNA expression of JAG-, DLL-, and NOTCH-DEGs is important for analyzing the relationship between JAG, DLL, and NOTCH families and clinicopathological data from cancer patients. Thus, our results, based on transcriptomic analysis with DEG enrichment, reflect JAG, DLL, and NOTCH activation and their clinical relevance.

**Response Figure 21 (S2B Fig in revised manuscript)** Box plots comparing the protein expressions of JAG, DLL, and NOTCH families in BIC, CRC, GBM, HNSC, KRCC, LHC, LGG, LUAD, OV, UCEC and the corresponding normal tissues. Data were analyzed using a two-tailed student’s t-test (**p* < 0.05; ***p* < 0.01; ****p* < 0.001).

**Response Figure 22 (Fig 1B in revised manuscript)** Box plots comparing the gene expressions of JAG, DLL, and NOTCH families in each cancer type and the corresponding normal tissues. Data were analyzed using a two-tailed student’s t-test (**p* < 0.05; ***p* < 0.01; ****p* < 0.001).

**Response Figure 23 (Fig 6A and S14 Fig in revised manuscript) A** A matrix demonstrating the overall result from the survival analysis using the expression levels or DEG enrichment scores of JAG, DLL, and NOTCH families in the datasets of each cancer type. **B** A matrix showing the overall result from the survival analysis using the positive and negative signatures. The difference in survival, of which the *p* value from the log-rank t-test is less than 0.05, was presented to cause worse or better outcome.

**Lines 227-233 of the revised manuscript.** “Most of JAG, DLL, and NOTCH families genes were lower in GBM than in normal brain tissue, however, the proteins were highly expressed in GBM than in normal brain tissue (Fig 1B and S2B Fig). In particular, both mRNA and protein expression of NOTCH2 was highly expressed only in LGG. The overall expression of JAG, DLL, and NOTCH families genes compared to normal tissues was distinguishable in each cancer. mRNA and protein expression of *JAG1*, *DLL1/4*, and *NOTCH1/4* were lower in LUAD than in normal lung tissue, on the other hand, in KRCC, all the JAG, DLL, and NOTCH families genes except *DLL3* were highly expressed (Fig 1B and S2B Fig).”

**Lines 516-522 of the revised manuscript.** “Survival analysis using expression and DEG enrichment demonstrated distinguishable results; for example, in KRCC, only *JAG1* expression led to better outcomes in patients with KRCC; however, the analysis with DEG enrichment showed that all the DEGs led to longer survival, except for the DLL3-DEGs (Fig 6A). In addition, *JAG1* expression did not affect clinical outcomes, whereas the enrichment of DEGs showed a negative impact on survival in GBM (Fig 6A). Similarly, survival analysis using the enrichment scores of the positive and negative signatures revealed the effect of the signatures on patient survival (S14 Fig).”

B. Results:

1. Explanation: In second paragraph of result section, author mentioned comparing normal tissue expression with cancer tissue, but they have not mentioned using normal tissue sample for study and datasets they have downloaded. Author should justify the reason and should mention the source of dataset collection.

**Response:** Thank you for your constructive feedback. The comparison of primary tumor and normal tissues was performed based on each TCGA dataset which included the transcriptomes of tumor samples and the corresponding normal tissues (**Response Figure 24A**). In addition, for BIC and OV, which did not include normal tissue samples, additional datasets were used for further comparison of the expression (**Response Figure 24B**). The datasets selected were restricted to those having more than 400 patient samples, allowing for a broad analysis. We have revised the manuscript to clearly state the reason for the selection and sources of dataset collection.

**Response Figure 24 (Fig 1B and S2A Fig in revised manuscript) A** Box plots comparing the gene expressions of JAG, DLL, and NOTCH families in each cancer type and the corresponding normal tissues. Data were analyzed using a two-tailed student’s t-test (**p* < 0.05; ***p* < 0.01; ****p* < 0.001). **B** Box plots comparing the gene expressions of JAG, DLL, and NOTCH families in BIC (GSE65194) and OV (GSE69428) and the corresponding normal tissues. Data were analyzed using a two-tailed student’s t-test (**p* < 0.05; ***p* < 0.01; ****p* < 0.001).

**Lines 101-105 of the revised manuscript.** “The comparison of primary tumor and normal tissue was performed based on each TCGA datasets which included the transcriptome of tumor sample and corresponding normal tissue. The datasets of BIC and OV from TCGA provided only tumor samples. To compare tumor and normal in BIC and OV, we obtained additional datasets with the accession numbers GSE65194 and GSE69428 curated by Gene Expression Omnibus (GEO).”

**Lines 222-225 of the revised manuscript.** “First, we compared the mRNA expression levels of the JAG, DLL, and NOTCH families in each cancer type and corresponding normal tissues (Fig 1B and S2A Fig). The results showed a diverse tendency of expression across cancer types; their expression was either higher or lower than that in normal tissues.”

2. In the 5th paragraph under the subheading “JAG, DLL, and NOTCH family’s expression tend to vary across the cancer types”, using proteomics datasets/protein expression would help the author to coordinate the JAG, DLL, and NOTCH families regulation in cancer.

**Response:** Thank you for your insightful comment. The JAG, DLL, and NOTCH family proteins undergo proteolytic cleavage, forming an intracellular domain that exhibits the transcriptional regulatory functions of target genes (5, 6, 16). These processes depend on ligand-receptor interactions in the canonical pathway (1, 2). However, in some cases, they are ligand-receptor interaction-independent (5, 6).

We compared the protein expression of the JAG, DLL, and NOTCH families in primary tumors and normal tissues in 10 cases (BIC, CTC, GBM, HNSC, KRCC, LGG, LHC, LUAD, OV, and UCEC) among the 15 cancer types (**Response Figure 25**). The criterion for selecting datasets was that the data should simultaneously include both primary tumor and normal tissue samples. The overall expression of JAG, DLL, and NOTCH families in tumor samples compared to normal tissues was distinguishable for each cancer type. For example, JAG1, NOTCH1, NOTCH2, and NOTCH3 were upregulated in GBM but downregulated in LUAD, whereas NOTCH3 was upregulated. In addition, a comparison of primary tumor and normal tissues at the transcriptomic and proteomic levels showed different tendencies according to cancer type (**Response Figure 25 and 26**). For example, in LUAD, primary tumors showed upregulated expression of JAG, DLL, and NOTCH family genes compared to normal tissues at both the transcriptional and translational levels. In contrast, the transcriptome and proteome showed opposite trends in GBM.

JAG, DLL, and NOTCH family proteins undergo cleavage, translocate into the nucleus, and regulate the transcription of target genes (5, 15). Thus, the functional activation of the JAG, DLL, and NOTCH families may be reflected in the transcriptional activation of target genes. Our results also demonstrated that DEG enrichment of JAG, DLL, and NOTCH families was more closely related to patient clinical outcomes than gene expression alone (**Response Figure 27**).

In conclusion, the expression of JAG-, DLL-, and NOTCH-DEG at the transcriptional level and their enrichment may be important in analyzing the relationship of JAG, DLL, and NOTCH families with clinicopathological data from cancer patients.

**Response Figure 25 (S2B Fig in revised manuscript)** Box plots comparing the protein expressions of JAG, DLL, and NOTCH families in BIC, CRC, GBM, HNSC, KRCC, LHC, LGG, LUAD, OV, UCEC and the corresponding normal tissues. Data were analyzed using a two-tailed student’s t-test (**p* < 0.05; ***p* < 0.01; ****p* < 0.001).

**Response Figure 26 (Fig 1B in revised manuscript)** Box plots comparing the gene expressions of JAG, DLL, and NOTCH families in each cancer type and the corresponding normal tissues. Data were analyzed using a two-tailed student’s t-test (**p* < 0.05; ***p* < 0.01; ****p* < 0.001).

**Response Figure 27 (Fig 6A and S14 Fig in revised manuscript) A** A matrix demonstrating the overall result from the survival analysis using the expression levels or DEG enrichment scores of JAG, DLL, and NOTCH families in the datasets of each cancer type. **B** A matrix showing the overall result from the survival analysis using the positive and negative signatures. The difference in survival, of which the *p* value from the log-rank t-test is less than 0.05, was presented to cause worse or better outcome.

**Lines 227-233 of the revised manuscript.** “Most of JAG, DLL, and NOTCH families genes were lower in GBM than in normal brain tissue, however, the proteins were highly expressed in GBM than in normal brain tissue (Fig 1B and S2B Fig). In particular, both mRNA and protein expression of NOTCH2 was highly expressed only in LGG. The overall expression of JAG, DLL, and NOTCH families genes compared to normal tissues was distinguishable in each cancer. mRNA and protein expression of *JAG1*, *DLL1/4*, and *NOTCH1/4* were lower in LUAD than in normal lung tissue, on the other hand, in KRCC, all the JAG, DLL, and NOTCH families genes except *DLL3* were highly expressed (Fig 1B and S2B Fig).”

**Lines 516-522 of the revised manuscript.** “Survival analysis using expression and DEG enrichment demonstrated distinguishable results; for example, in KRCC, only *JAG1* expression led to better outcomes in patients with KRCC; however, the analysis with DEG enrichment showed that all the DEGs led to longer survival, except for the DLL3-DEGs (Fig 6A). In addition, *JAG1* expression did not affect clinical outcomes, whereas the enrichment of DEGs showed a negative impact on survival in GBM (Fig 6A). Similarly, survival analysis using the enrichment scores of the positive and negative signatures revealed the effect of the signatures on patient survival (S14 Fig).”

3. Clarification: why did not author performed cross-validation using independent datasets to prevent overfitting and conduct subgroup analysis to account for patient heterogeneity which could have decreased the inconsistency in correlations observed.

**Response:** Thank you for your suggestion. To investigate the function and clinical relevance of the JAG, DLL, and NOTCH families, we utilized patient expression datasets containing more than 400 samples including paired normal tissues. The overall expression of the JAG, DLL, and NOTCH families in tumor samples compared to that in normal tissues showed diverse trends according to the cancer type. We also analyzed the JAG, DLL, and NOTCH families using proteomic and single-cell datasets.

Although cross-validation using an independent cohort of transcriptomic data was not performed, selecting a large cohort containing mRNA expression data with corresponding clinicopathological information enabled transcriptomic analysis and its correlation with patient outcome. In addition, a wide-range analysis was possible with a large number of patients.

In addition, we performed a transcriptomic analysis using two independent single-cell sequencing datasets of GBM (**Response Figure 28 and 29**). Clusters highly expressing JAG or NOTCH families showed enriched hallmark signatures, including KRAS signaling and hypoxia. These results correspond to the finding that the combination of gene expression or DEG enrichment of JAG and NOTCH families with KRAS signaling or hypoxia signatures correlated with patient outcomes. Thus, the similarity of the analyses at the tumor bulk and single-cell levels suggested that our extensive analyses were reproducible.

**Response Figure 28 (Fig 4A, 4B and 4D in revised manuscript)** Expression of JAG, DLL, and NOTCH families and enrichment of positive and negative signatures using scRNA-seq of GBM. **A** tSNE plot of GBM cells colored by cluster. **B** Log-normalized expression of JAG, DLL, and NOTCH families. **C** Heatmap showing the enrichment of positive and negative signatures in each cluster.

**Response Figure 29 (S9D-F Fig in revised manuscript)** DEG-based GSEA of hallmark using scRNA-seq in GBM (GSE176278). **A** tSNE plot of GBM cells colored by cluster. **B** Log-normalized expression of JAG, DLL, and NOTCH families. **C** Heatmap showing the enrichment of positive and negative signatures in each cluster.

**Lines 388-391 of the revised manuscript.** “To directly verify the correlations between the JAG, DLL, and NOTCH families and the signatures demonstrated by *in silico* analysis, we analyzed the scRNA-seq data of GBM (24). Visualization of GBM cells using tSNE revealed extremely high heterogeneity, and the cells were divided into 13 clusters (Fig 4A).”

**Lines 401-406 of the revised manuscript.** “The expression of the JAG, DLL, and NOTCH families was characterized in each cluster (Fig 4B and S7A Fig). The JAG family exhibited high expression in cluster 10, whereas the DLL family showed no specific cluster with high expression. *JAG1* was highly expressed in clusters 2, 3, 5, and 10, with the highest expression observed in cluster 5. Except for *NOTCH4*, all NOTCH family members were commonly expressed in clusters 0, 1, and 6. Members of the JAG, DLL, and NOTCH families were highly expressed in distinct clusters.”

**Lines 416-421 of the revised manuscript.** “GSEA demonstrated that clusters 5 and 10 were enriched with the signatures belonging to the positive signatures, such as KRAS signaling UP, UV response UP, and hypoxia (Fig 4D). However, most positive signatures were enriched in cluster 0, including EMT, myogenesis, hypoxia, KRAS signaling UP, NOTCH signaling, and IL2-STAT5 signaling (Fig 4D). While the NOTCH family regulates major biological processes, the JAG and DLL families regulate only a small fraction of the biological process, but they are independent.”

**Lines 422-425 of the revised manuscript.** “Additionally, we analyzed scRNA-seq data of BIC, GBM, and LHC to verify consistency at various cancer types (S8 and S9 Fig) (24, 31, 32). There were no clusters in which the JAG, DLL, and NOTCH families were individually expressed. Among Notch ligands, *JAG1* was most expressed in many clusters, and expression of receptors showed different patterns across cancer types.”

4. Justification: Author analysis single cell sequencing data which provides only a snapshot. But Heterogeneity within tumour samples was not addressed, performing spatial and temporal modelling with multi-region sequencing and circulating tumour DNA data will strengthen the analysis.

**Response:** Thank you for your insightful comment. Our results indicated that enrichment of JAG, DLL, and NOTCH families-related hallmark signatures investigated in bulk-sequencing data was reproduced at the single-cell level (**Response Figure 30-32**). For instance, the correlation between the JAG or NOTCH family and hallmarks such as KRAS signaling and hypoxia were consistently observed in two independent datasets of GBM (**Response Figure 30 and 32D-F)**. These results indicated that the high expression or activity of the JAG, DLL, and NOTCH families was related to specific signatures. In addition, our goal was to demonstrate the signatures enriched in JAG, DLL, and NOTCH upregulated or activated cell populations and to suggest a possible clinicopathological relationship irrelevant to temporal fluctuation or spatial distribution. Thus, we have revised our manuscript and included supplementary comments on the single-cell data analysis.

**Response Figure 30 (Fig 4A, 4B and 4D in revised manuscript)** Expression of JAG, DLL, and NOTCH families and enrichment of positive and negative signatures using scRNA-seq of GBM. **A** tSNE plot of GBM cells colored by cluster. **B** Log-normalized expression of JAG, DLL, and NOTCH families. **C** Heatmap showing the enrichment of positive and negative signatures in each cluster.

**Response Figure 31 (S8 Fig in revised manuscript)** DEG-based GSEA of hallmark using scRNA-seq in LHC (GSE125449). **A** tSNE plot of LHC cells colored by cluster. **B** Log-normalized expression of JAG, DLL, and NOTCH families. **C** Heatmap showing the expression of JAG, DLL, and NOTCH families in each cluster. **D** Heatmap showing the enrichment of hallmark signatures in each cluster.

**Response Figure 32 (S9 Fig in revised manuscript)** DEG-based GSEA of hallmark using scRNA-seq in BIC (GSE195665) and GBM (GSE176278). **A, D** tSNE plot of (A) BIC and (D) GBM cells colored by cluster. **B**, **E** Log-normalized expression of JAG, DLL, and NOTCH families in (B) BIC and (E) GBM cells**.** **C, F** Heatmap showing the expression of JAG, DLL, and NOTCH families in each cluster of (C) BIC and (F) GBM.

**Lines 388-391 of the revised manuscript.** “To directly verify the correlations between the JAG, DLL, and NOTCH families and the signatures demonstrated by *in silico* analysis, we analyzed the scRNA-seq data of GBM (24). Visualization of GBM cells using tSNE revealed extremely high heterogeneity, and the cells were divided into 13 clusters (Fig 4A).”

**Lines 401-406 of the revised manuscript.** “The expression of the JAG, DLL, and NOTCH families was characterized in each cluster (Fig 4B and S7A Fig). The JAG family exhibited high expression in cluster 10, whereas the DLL family showed no specific cluster with high expression. *JAG1* was highly expressed in clusters 2, 3, 5, and 10, with the highest expression observed in cluster 5. Except for *NOTCH4*, all NOTCH family members were commonly expressed in clusters 0, 1, and 6. Members of the JAG, DLL, and NOTCH families were highly expressed in distinct clusters.”

**Lines 416-421 of the revised manuscript.** “GSEA demonstrated that clusters 5 and 10 were enriched with the signatures belonging to the positive signatures, such as KRAS signaling UP, UV response UP, and hypoxia (Fig 4D). However, most positive signatures were enriched in cluster 0, including EMT, myogenesis, hypoxia, KRAS signaling UP, NOTCH signaling, and IL2-STAT5 signaling (Fig 4D). While the NOTCH family regulates major biological processes, the JAG and DLL families regulate only a small fraction of the biological process, but they are independent.”

**Lines 422-425 of the revised manuscript.** “Additionally, we analyzed scRNA-seq data of BIC, GBM, and LHC to verify consistency at various cancer types (S8 and S9 Fig) (24, 31, 32). There were no clusters in which the JAG, DLL, and NOTCH families were individually expressed. Among Notch ligands, *JAG1* was most expressed in many clusters, and expression of receptors showed different patterns across cancer types.”

Minor comments:

1. There are many typing and grammatical errors, throughout the whole paper including figure and figure legends.

We apologize for our mistakes. We have revised our manuscript to clarify and correct typos.

2. Few abbreviations with the full forms are not mentioned.

We apologize for the inconvenience caused. We have revised our manuscript to include the full names of the abbreviations mentioned for the first time.

3. Figure legends should provide more details.

We have enhanced the clarity of the figure legends to make them clearer.

4. The lack of description for why certain analyses were chosen leaves the rationale unclear.

We have revised our manuscript with more descriptions because each analysis was performed to support our rationale more clearly. In summary, analyses were conducted to explore the correlation between clinicopathology and the expression of JAG, DLL, and NOTCH families as well as their DEG enrichment. Hallmark signature enrichment was analyzed to obtain insights into the phenotypic features related to JAG, DLL, and NOTCH families. Ultimately, analyses of patient prognosis based on JAG, DLL, and NOTCH expression, DEG enrichment, and signature enrichment were conducted to analyze the clinical relevance and propose potential phenotypes as targets in JAG, DLL, or NOTCH-targeted therapy.

References

1.	Hoey T, Yen WC, Axelrod F, Basi J, Donigian L, Dylla S, et al. DLL4 blockade inhibits tumor growth and reduces tumor-initiating cell frequency. Cell Stem Cell. 2009;5(2):168-77.

2.	Leong KG, Niessen K, Kulic I, Raouf A, Eaves C, Pollet I, et al. Jagged1-mediated Notch activation induces epithelial-to-mesenchymal transition through Slug-induced repression of E-cadherin. J Exp Med. 2007;204(12):2935-48.

3.	Yang L, Shi P, Zhao G, Xu J, Peng W, Zhang J, et al. Targeting cancer stem cell pathways for cancer therapy. Signal Transduct Target Ther. 2020;5(1):8.

4.	Hu Y, Su H, Li X, Guo G, Cheng L, Qin R, et al. The NOTCH ligand JAGGED2 promotes pancreatic cancer metastasis independent of NOTCH signaling activation. Mol Cancer Ther. 2015;14(1):289-97.

5.	Kim JY, Hong N, Park S, Ham SW, Kim EJ, Kim SO, et al. Jagged1 intracellular domain/SMAD3 complex transcriptionally regulates TWIST1 to drive glioma invasion. Cell Death Dis. 2023;14(12):822.

6.	Pelullo M, Nardozza F, Zema S, Quaranta R, Nicoletti C, Besharat ZM, et al. Kras/ADAM17-Dependent Jag1-ICD Reverse Signaling Sustains Colorectal Cancer Progression and Chemoresistance. Cancer Res. 2019;79(21):5575-86.

7.	Hu J, Yu J, Gan J, Song N, Shi L, Liu J, et al. Notch1/2/3/4 are prognostic biomarker and correlated with immune infiltrates in gastric cancer. Aging (Albany NY). 2020;12(3):2595-609.

8.	Qing Y, Wang Y, Hu C, Zhang H, Huang Y, Zhang Z, et al. Evaluation of NOTCH family genes' expression and prognostic value in prostate cancer. Transl Androl Urol. 2022;11(5):627-42.

9.	Yousefi H, Bahramy A, Zafari N, Delavar MR, Nguyen K, Haghi A, et al. Notch signaling pathway: a comprehensive prognostic and gene expression profile analysis in breast cancer. BMC Cancer. 2022;22(1):1282.

10.	Zhang Y, Shang L, Han J, Shen X, Liu H, Yang J, et al. Biological and immunological significance of DLL3 expression in different tumor tissues: a pan-cancer analysis. Aging (Albany NY). 2023;15(9):3427-41.

11.	Cerami E, Gao J, Dogrusoz U, Gross BE, Sumer SO, Aksoy BA, et al. The cBio cancer genomics portal: an open platform for exploring multidimensional cancer genomics data. Cancer Discov. 2012;2(5):401-4.

12.	Bowman RL, Wang Q, Carro A, Verhaak RG, Squatrito M. GlioVis data portal for visualization and analysis of brain tumor expression datasets. Neuro Oncol. 2017;19(1):139-41.

13.	Maubant S, Tesson B, Maire V, Ye M, Rigaill G, Gentien D, et al. Transcriptome analysis of Wnt3a-treated triple-negative breast cancer cells. PLoS One. 2015;10(4):e0122333.

14.	Yamamoto Y, Ning G, Howitt BE, Mehra K, Wu L, Wang X, et al. In vitro and in vivo correlates of physiological and neoplastic human Fallopian tube stem cells. J Pathol. 2016;238(4):519-30.

15.	Borggrefe T, Oswald F. The Notch signaling pathway: transcriptional regulation at Notch target genes. Cell Mol Life Sci. 2009;66(10):1631-46.

16.	Aster JC, Pear WS, Blacklow SC. The Varied Roles of Notch in Cancer. Annu Rev Pathol. 2017;12:245-75.
