# Supplementary material for: Cancer-wide in silico analyses using differentially expressed genes demonstrate the functions and clinical relevance of JAG, DLL, and NOTCH
Source: PLoS One. 2024 Jul 29;19(7):e0307943. doi: 10.1371/journal.pone.0307943 (PMC11285958; doi:10.1371/journal.pone.0307943)
Supplement: Attachment — Submitted filename: 4. Response to Reviewers_Kim et al.docx [file pone.0307943.s003.docx]

**A rebuttal letter for revision of PONE-D-23-37633R2**

**Cancer-wide in silico analyses using differentially expressed genes demonstrate the functions and clinical relevance of *JAG*, *DLL*, and *NOTCH***

Jung Yun Kim et al.

**Reviewer #1:** It is suggested that after pooled analysis, the results of JAG, DLL, and NOTCH gene expression and clinicopathological features in common solid tumors can be highlighted and presented separately, not only focusing on GMB, LHC and HNSC.

**Response:** Thank you for your insightful comment. The expression of JAG, DLL, and NOTCH families is linked with clinicopathological features including tumor stage progression, metastatic spread, therapeutic response, and recurrence in several tumor types. As DEG enrichment rather than the expression of JAG, DLL, and NOTCH families showed significant correlation with the positive or negative signature of JAG, DLL, and NOTCH families, we performed additional analyses on the linkage between DEG enrichment and clinicopathological phenotypes. The analyses include JAG, DLL, and NOTCH families’ DEG enrichment according to tumor stage, primary/metastasis, pre-/post-chemotherapy, and primary/recurrent status of tumors.

First, we investigated the DEG enrichment of JAG, DLL, and NOTCH families according to the tumor stage. While the mRNA expression of JAG, DLL, and NOTCH families showed a correlation with tumor stage in a few tumor types, DEG enrichment of each families showed a significant correlation in most tumors (**Response Figure 1 and 2**). These results correspond to DEG enrichment showing a more significant relationship with positive or negative signatures of JAG, DLL, and NOTCH families than mRNA expression of JAG, DLL, and NOTCH families (**Response Figure 3**).

Interestingly, in GBM, DEG enrichment of ligands except JAG1 and DLL4, which increased stage-dependently, decreased according to tumor stage (**Response Figure 2A-E**). On the other hand, the enrichment of DEG of all NOTCH receptors showed a stage-dependent increase (**Response Figure 3F-I**). Especially, NOTCH1-DEG enrichment increased significantly despite the decrease in mRNA expression of NOTCH1 according to tumor stage (**Response Figure 1F and 2F**). These results might be due to the ligand-dependent signaling activation of NOTCH1 by elevated JAG1 and DLL4 (1, 2). Furthermore, among 1,023 and 1,288 genes up-regulated by transcriptional activity of the intracellular domain of JAG1 and NOTCH1 respectively, 487 genes were commonly regulated by JAG1 and NOTCH1 in our previous study (3). As DEG of Notch ligand and receptor highly overlaps, upregulated JAG1 and DLL4 might have driven these results.

For KRCC, only DLL3-DEG increased stage-dependently while DEG of other ligands and receptors decreased (**Response Figure 2**). Correspondingly, enrichment of DLL3-DEG is correlated with poor clinical outcome while DEG of other ligands and receptors showed better outcomes (**Response Figure 4**). In addition, the DEG of JAG and DLL families increased in a stage-dependent manner in LHC (**Response Figure 2**). These results might indicate the intact relationship between NOTCH signaling and tumor progression in various tumors. Also, various trends of DEG enrichment according to tumor stage for each gene of JAG, DLL, and NOTCH families and tumor type might be due to the diversity of composition and expressions of DEG for each gene and tumor.

Second, we compared DEG enrichment of JAG, DLL, and NOTCH families in primary and metastatic tumors. While the expression of JAG, DLL, and NOTCH families showed differences in primary and metastatic tumors, DEG enrichment differed only in a few tumors (**Response Figures 5, 6 and 7**). Although DEG enrichment was more closely related to the tumor stage than the expression, metastasis was not the case. Moreover, although the expression of JAG1 decreased, enrichment of JAG1-DEG increased in metastatic PRAD compared to primary tumors (**Response Figures 5A and 6A**). As a Notch signal depends on cell-cell interaction, its activation and function are in a context-dependent manner. Interestingly, the expression of transcriptional targets of the Notch signal showed different patterns in primary and metastatic sites in the previous study (4). DEG, which we established, was selected based on separated groups of patients with high or low expression of each JAG, DLL, and NOTCH family gene in primary tumors. Thus, the results on DEG enrichment in metastatic tumors may not be able to represent the context-dependent effect of Notch signal in metastatic tumors.

In addition, we performed an analysis between samples from patients before and after therapy (**Response Figures 8 and 9**). There were no changes in DEG enrichment in CRC, KRCC, and LHC (**Response Figures 9B, 9D, and 9E**). For SCC, DEG enrichment of JAG1, JAG2, and NOTCH2 changed after therapy although the expression of all JAG, DLL, and NOTCH families did not show any difference (**Response Figures 8F and 9F**). The patterns of gene expression and DEG enrichment were similar only in BIC and HNSC. Both the expression and DEG enrichment of JAG1, DLL1, and NOTCH4 significantly increased after therapy in BIC (**Response Figures 8C and 9A**). JAG2, NOTCH1, and NOTCH3 tend to increase after therapy, although not significant. Interestingly, only DLL3 decreased after therapy in BIC. In HNSC, the expression and DEG enrichment of JAG1, DLL1, and NOTCH2 increased after therapy, while DLL3 and NOTCH4 decreased (**Response Figures 8B and 9C**). However, the expression of NOTCH3 and its DEG enrichment showed opposite trends. These results might be due to that DEG enrichment can be significantly changed depending on the activation or inhibition of other signals that interact with the Notch signaling after anti-cancer therapy (5).

We also compared DEG enrichment in primary and recurrent tumors (**Response Figures 10 and 11**). BIC, LGG, and GBM showed a change in DEG enrichment between primary and recurrent tumors. Among Notch ligands, only DLL3-DEG enrichment decreased in recurrent BIC (**Response Figure 10**). Also, DEG enrichment of JAG1, JAG2, and DLL1 decreased in recurrent GBM (**Response Figure 10**). In the case of Notch receptors, DEG enrichment of NOTCH1 and NOTCH2 in BIC, NOTCH4 in GBM, and NOTCH2 in LGG decreased in recurrent tumors (**Response Figure 11**). The outcome of Notch signal activation may differ from crosstalk with multiple signaling pathways in cancer (6). Also, recurrent tumors show dissimilar gene expression and signaling pathway components compared to primary tumors (7). As DEG, which we established, was selected based on primary tumors, DEG enrichment may not be appropriate to explain the context-dependent effect of Notch signal in recurrent tumors.

Collectively, these results indicate that DEG related to Notch signal, in addition to the expression of JAG, DLL, and NOTCH families, might give insights into tumor progression and clinical outcome. In addition, further investigation on the context-dependent change of DEG of JAG, DLL, and NOTCH families might explain the linkage of the Notch signal and pathological phenotypes of cancer.

**Response Figure 2 (S4 Fig in revised manuscript)** Comparison of enrichment score of DEG for each JAG, DLL, and NOTCH families according to tumor stage. Box plots showing the enrichment score of (A) JAG1 DEG, (B) JAG2 DEG, (C) DLL1 DEG, (D) DLL3 DEG, (E) DLL4 DEG, (F) NOTCH1 DEG, (G) NOTCH2 DEG, (H) NOTCH3 DEG, and (I) NOTCH4 DEG according to tumor stage.

Response Figure 1 (S3 Fig in revised manuscript) Box plots showing the gene expressions of (A) JAG1, (B) JAG2, (C) DLL1, (D) DLL3, (E) DLL4, (F) NOTCH1, (G) NOTCH2, (H) NOTCH3, and (I) NOTCH4 according to tumor stage.

**Response Figure 4 (Fig 6A in revised manuscript)** A matrix demonstrating the overall result from the survival analysis using the expression levels or DEG enrichment scores of JAG, DLL, and NOTCH families in the datasets of each cancer type.

**Response Figure 3 (Fig 3B in revised manuscript)** Heatmap showing the correlation between the hallmark signatures and expression levels (top) or DEG enrichment scores (bottom) of the JAG, DLL, and NOTCH families.

**Response Figure 5 (Fig 5A and S11 Fig in revised manuscript)** Box plots comparing the expression of (A) JAG1, (B) JAG2, (C) DLL1, (D) DLL3, (E) DLL4, (F) NOTCH1, (G) NOTCH2, (H), NOTCH3, and (I) NOTCH4 in primary and metastatic tumor. Data were analyzed using a two-tailed student’s t-test (**p* < 0.05, ***p* < 0.01, ****p* < 0.001)

**Response Figure 6 (S15 Fig in revised manuscript)** Box plots showing the enrichment score of (A) JAG1 DEG, (B) JAG2 DEG, (C) DLL1 DEG, (D) DLL3 DEG, and (E) DLL4 DEG in primary and metastatic tumors. Data were analyzed using a two-tailed Student’s t-test (**p* < 0.05, ***p* < 0.01, ****p* < 0.001).

**Response Figure 7 (S16 Fig in revised manuscript)** Box plots showing the enrichment score of (A) NOTCH1 DEG, (B) NOTCH2 DEG, (C) NOTCH3 DEG, and (D) NOTCH4 DEG in primary and metastatic tumors. Data were analyzed using a two-tailed Student’s t-test (**p* < 0.05, ****p* < 0.001).

**Response Figure 8 (Fig 5B, 5C, and S12 Fig in revised manuscript) (**A) Box plots comparing the expression of JAG, DLL, and NOTCH families in before and after chemotherapy with IFNa-2b in LHC. Data were analyzed using a two-tailed student’s t-test (**p* < 0.05; ***p* < 0.01). (B) Box plots comparing the expression of JAG, DLL, and NOTCH families in before and after radiotherapy and chemotherapy with cetuximab and AMG479 in HNSC. Data were analyzed using a two-tailed student’s t-test (**p* < 0.05; ***p* < 0.01; ****p* < 0.001). (C) The expression of JAG, DLL, and NOTCH families in before and after chemotherapy in BIC. Data were analyzed using a two-tailed student’s t-test (**p* < 0.05; ****p* < 0.001). (D) The expression of JAG, DLL, and NOTCH families in before and after chemotherapy in CRC. (E) The expression of JAG, DLL, and NOTCH families in before, 3 months and 6 months after chemotherapy with pazopanib in KRCC. Data were analyzed using a two-tailed student’s t-test (**p* < 0.05). (F) The expression of JAG, DLL, and NOTCH families in before and after chemotherapy with dabrafenib or vemurafenib in SCC.

**Response Figure 9 (S17 Fig in revised manuscript)** Box plots comparing the enrichment score of DEG for each JAG, DLL, and NOTCH families in patients with anti-cancer drugs. (A) Enrichment score of DEG for each JAG, DLL, and NOTCH families before and after chemotherapy in BIC. Data were analyzed using a two-tailed Student’s t-test (**p* < 0.05, ****p* < 0.001). (B) Enrichment score of DEG for each JAG, DLL, and NOTCH families before and after chemotherapy in CRC. (C) Enrichment score of DEG for each of JAG, DLL, and NOTCH families before and after radiotherapy and chemotherapy with cetuximab and AMG479 in HNSC. (D) Enrichment score of DEG for each JAG, DLL, and NOTCH families before, 3 months, and 6 months after chemotherapy with pazopanib in KRCC. (E) Enrichment score of DEG for each JAG, DLL, and NOTCH families before and after chemotherapy with IFNa-2b in LHC. (F) Enrichment score of DEG for each JAG, DLL, and NOTCH families before and after chemotherapy with dabrafenib or vemurafenib in SCC. Data were analyzed using a two-tailed Student’s t-test (**p* < 0.05).

**Response Figure 10 (S18 Fig in revised manuscript)** Tracking enrichment score of DEG for each Notch ligand between primary and recurrent tumors of each patient with BIC, GBM, HNSC, LGG, LHC, OV, SCC, and TC. Scatterplots including (A) JAG1 DEG, (B) JAG2 DEG, (C) DLL1 DEG, (D) DLL3 DEG, and (E) DLL4 DEG.

**Response Figure 11 (S19 Fig in revised manuscript)** Tracking enrichment score of DEG for each Notch receptor between primary and recurrent tumors of each patient with BIC, GBM, HNSC, LGG, LHC, OV, SCC, and TC. Scatterplots including (A) NOTCH1 DEG, (B) NOTCH2 DEG, (C) NOTCH3 DEG, and (D) NOTCH4 DEG.

**Lines 284-303 of the revised manuscript.** “Next, we compared the enrichment score of DEG for each JAG, DLL, and NOTCH families according to the tumor stage (S4 Fig). The expression of JAG, DLL, and NOTCH families was correlated with tumor stage in a few cancer types, while each DEG enrichment was significantly associated with most cancers (S3 and S4 Fig).

Interestingly, the DEG enrichment of JAG1, DLL4, and all Notch receptors increased according to tumor stage (S4 Fig). NOTCH1-DEG enrichment increased significantly despite the decrease in mRNA expression of NOTCH1 according to tumor stage (S3 and S4 Fig). The transcriptional DEGs of JAG1 and NOTCH1 overlap significantly according to our previous results (29). Our results might be due to the ligand-dependent signaling activation of NOTCH1 by abundant JAG1 and DLL4. Furthermore, because DEG of Notch ligands and receptors highly overlap, upregulated JAG1 and DLL4 might have driven these trends.

For KRCC, DEG enrichment of most JAG, DLL, and NOTCH families decreased stage-dependently while only DLL3-DEG enrichment increased (S4 Fig). These results can be expected that DLL3 may have an inverse effect on KRCC as opposed to others. In addition, DEG of all Notch ligands increased in stage-dependent manner in LHC.

The diversity of the composition and expression of DEGs for each gene and tumor influenced the different trends in DEG enrichment for each gene. Moreover, the Notch signal interacts with other oncogenic signals, and they also affect the regulation of the expression of target genes of the Notch signal (31, 32). Since the signaling mechanisms activated in each tumor are different, the aspect of their DEG enrichment is observed in various trends (33, 34).”

**Lines 503-539 of the revised manuscript.** “To confirm the relationship between DEG for each gene and clinicopathological features in common solid tumors, we performed additional analyses to compare DEG enrichment with four different clinicopathological features including tumor stage, metastasis, recurrent, and post-chemotherapy status of tumors.

Expression of JAG, DLL, and NOTCH families between primary and metastatic tumors differed in most cancers, and DEG enrichment was more closely related to tumor stage than the expression of ligands and receptors (Fig 5A, S3, S4, S11, S15, and S16 Fig). However, DEG enrichment between primary and metastatic tumors showed differences only in a few. Even in the case of PRAD, the expression of JAG1 and JAG1-DEG enrichment were reversed. The expression of JAG1 decreased but DEG enrichment increased in metastatic tumors (Fig 5A and S15A).

For each cancer type, we compared the DEG enrichment of each gene between samples from patients before and after anti-cancer therapy (S17 Fig). Gene expression and DEG enrichment are similar only in BIC and HNSC (S17A and S17C). There were no differences in DEG enrichment for each gene before and after anti-cancer therapy in CRC, KRCC, and LHC (S17B, S17D, and S17E Fig).

Both expression and DEG enrichment of JAG1, DLL1, and NOTCH4 significantly increased after anti-cancer therapy in BIC (S17A Fig). In addition, JAG2, NOTCH1, and NOTCH3 tend to increase after anti-cancer therapy, although there is no significance. Interestingly, DLL3 decreased after anti-cancer therapy, contrary to DEG enrichment of other genes.

In HNSC, both expression and DEG enrichment of JAG1, DLL1, and NOTCH2 significantly increased after anti-cancer therapy, while DLL3 and NOTCH4 significantly decreased (S17C Fig). However, NOTCH3 expression and NOTCH3-DEG enrichment showed opposite trends in HNSC. There were no differences in all gene expression before and after anti-cancer therapy in SCC, but JAG1-, JAG2-, and NOTCH2-DEG enrichment significantly increased or decreased after anti-cancer therapy (S17F Fig). DEG enrichment can be significantly changed depending on the activation or inhibition of other signals that interact with the Notch signaling after anti-cancer therapy (31, 32).

We compared the DEG enrichment in primary and recurrent tumors (S18 and S19 Fig). Changes in DEG enrichment between primary and recurrent tumors were limited to BIC, LGG, and GBM. In Notch ligands, only DLL3-DEG enrichment decreased in recurrent BIC and JAG1-, JAG2-, and DLL1-DEG enrichment decreased in recurrent GBM (S18 Fig). DEG enrichment of Notch receptors was also similar (S19 Fig). NOTCH1- and NOTCH2-DEG enrichment decreased in recurrent BIC. NOTCH4-DEG enrichment decreased in recurrent GBM and NOTCH2 DEG decreased in recurrent LGG.

Notch signal is a cell-cell interaction-dependent signaling mechanism, known to regulate gene expression in a cell-type and context-dependent manner. Notch signal promotes and inhibits tumor development in a context-dependent manner (33, 34). In addition, the outcomes of Notch signal can be altered as Notch receptors crosstalk with other signal receptors in the tumor microenvironment. Because our DEGs for each gene were selected based on the primary tumor, the enrichment of their DEGs cannot confirm a context-dependent effect in metastatic or recurrent tumors.”

**Lines 576-577 of the revised manuscript.** “Not only did DLL3 show the opposite DEG enrichment tendency to others but its hallmarks enrichment tendency and effect on patient survival also showed an opposite trend (Fig 3B, 6A, S4 Fig).

**Reviewer #3:** The authors has done a wonderful job while addressing the comments raised in a previous round of review. In summary, this study is a remarkable contribution to cancer biology, providing extensive insights into the multifaceted roles of Notch signaling. The integration of bioinformatics with experimental data offers a powerful approach to unraveling the complexities of cancer progression and therapeutic resistance. This research not only advances our understanding of Notch signaling but also paves the way for developing more effective cancer treatments.

**Response:** Thank you very much for your kind review. In the present study, we demonstrated that JAG, DLL, and NOTCH families gene expression and their DEG vary among tumor types and that they might regulate diverse cellular physiology. We also investigated the cancer-wide function of JAG, DLL, and NOTCH families and discussed the linkage with clinicopathological features including tumor progression, metastasis, therapeutic response, and therapy resistance in tumors. As you mentioned, our results may provide insights into the diverse role of Notch signaling in tumor progression and therapeutic resistance. A comprehensive investigation of Notch signaling and associated biological features would support further understanding of the regulatory mechanism of malignancy and, therefore might provide the way to the most efficient therapeutic approach.

References

1.	Zhang X, Chen T, Zhang J, Mao Q, Li S, Xiong W, et al. Notch1 promotes glioma cell migration and invasion by stimulating β-catenin and NF-κB signaling via AKT activation. Cancer Sci. 2012;103(2):181-90.

2.	Purow BW, Haque RM, Noel MW, Su Q, Burdick MJ, Lee J, et al. Expression of Notch-1 and its ligands, Delta-like-1 and Jagged-1, is critical for glioma cell survival and proliferation. Cancer Res. 2005;65(6):2353-63.

3.	Kim EJ, Kim JY, Kim SO, Hong N, Choi SH, Park MG, et al. The oncogenic JAG1 intracellular domain is a transcriptional cofactor that acts in concert with DDX17/SMAD3/TGIF2. Cell Rep. 2022;41(8):111626.

4.	Kondratyev M, Pesic A, Ketela T, Stickle N, Beswick C, Shalev Z, et al. Identification of acquired Notch3 dependency in metastatic Head and Neck Cancer. Commun Biol. 2023;6(1):538.

5.	Wang WM, Zhao ZL, Ma SR, Yu GT, Liu B, Zhang L, et al. Epidermal growth factor receptor inhibition reduces angiogenesis via hypoxia-inducible factor-1α and Notch1 in head neck squamous cell carcinoma. PLoS One. 2015;10(2):e0119723.

6.	Misiorek JO, Przybyszewska-Podstawka A, Kałafut J, Paziewska B, Rolle K, Rivero-Müller A, et al. Context Matters: NOTCH Signatures and Pathway in Cancer Progression and Metastasis. Cells. 2021;10(1).

7.	Schäfer N, Gielen GH, Rauschenbach L, Kebir S, Till A, Reinartz R, et al. Longitudinal heterogeneity in glioblastoma: moving targets in recurrent versus primary tumors. J Transl Med. 2019;17(1):96.
